# Defining the heterogeneous molecular landscape of lung cancer cell responses to epigenetic inhibition

**DOI:** 10.1038/s42003-025-09455-0

**Published:** 2026-01-28

**Authors:** Chuwei Lin, Catherine M. Sniezek, Christopher D. McGann, Rashmi Karki, Ross M. Giglio, Benjamin A. Garcia, José L. McFaline-Figueroa, Devin K. Schweppe

**Affiliations:** 1 Genome Sciences, University of Washington, Seattle, WA USA; 2 Department of Biochemistry and Molecular Biophysics, Washington University School of Medicine, St. Louis, MO USA; 3 Biomedical Engineer, Columbia University, New York, NY USA; 4 Brotman Baty Institute for Precision Medicine, Seattle, WA USA; 5 Institute of Stem Cell and Regenerative Medicine, University of Washington, Seattle, WA USA

**Keywords:** Proteomics, Proteomics

## Abstract

Epigenetic inhibitors exhibit powerful antiproliferative and anticancer activities. However, cellular responses to small-molecule epigenetic inhibition are heterogeneous and dependent on factors such as the genetic background and metabolic state of cells, as well as on-/off-target engagement of individual small-molecule compounds. The molecular study of the extent of this heterogeneity often measures changes in a single cell line. To more comprehensively profile the effects of small-molecule perturbations and their influence on heterogeneous cellular responses, we present a molecular resource based on the quantification of chromatin, proteome, and transcriptome remodeling due to histone deacetylase inhibitors (HDACi) in non-isogenic cell lines. Through quantitative molecular profiling of 10,621 proteins, these data reveal coordinated molecular remodeling of HDACi treated cancer cells. HDACi-regulated proteins differ greatly across cell lines with consistent (JUN, MAP2K3, CDKN1A) and divergent (CCND3, ASF1B, BRD7) cell-state effectors. Together these data provide valuable insight into cell-type driven and heterogeneous responses that must be taken into consideration when monitoring molecular perturbations in culture models. We have also built a web interface for the extensive amount of data to allow users to explore the data as a resource for understanding chemical perturbation of diverse cell types.

## Abstract (web summary)

Quantitative multi-omics profiling of HDAC inhibitor responses in non-isogenic cancer cell lines reveals shared and divergent molecular remodeling. Responses highlight cell-type-dependent and -independent epigenetic drug mechanisms of action.

## Introduction

Deconvoluting the myriad effects downstream of small molecule perturbations is essential to therapeutic development. Whole proteome and transcriptome analyses of these perturbations can validate and identify proposed mechanisms of action and identify cellular effects for therapeutics and tool compounds. To date, however, many large-scale proteome analyses of drug perturbations have been limited to a single cell line treated with a cohort of small-molecule tool compounds and therapeutic drugs (Supplementary table 1). The selection of cell lines in these studies has been in part a practical consideration as each new cell line included leads to a rapid expansion in the number of samples that must be processed and compared. Unfortunately, this often means sacrificing understanding of the diverse molecular contexts of different cellular models to enable the analysis of larger cohorts of small-molecule compounds^1^.

Large-scale viability screening of small molecule perturbations in the PRISM dataset has shown that drugs targeting the same protein or pathway can drive heterogeneous responses in cancer cells^2^. Recent transcriptome efforts for bulk and single-cell samples have also uncovered divergent cellular responses when cells are treated with therapeutic and tool compounds^3^. At the single-cell level, these studies revealed inconsistent molecular responses of individual, isogenic cells to small molecule perturbation suggesting that additional context and molecular understanding are necessary to dissect the molecular consequences of chemical perturbation. More challenging still is the fact that transcriptomic profiling of cellular perturbations cannot measure the direct protein targets of many of these perturbations^4^. Thus, owing to the limited correlation between RNA and protein measurements^5^, there remains a gap in our knowledge of the proteomic consequences of drug perturbations across multiple, representative cell lines.

Recently, several studies have aimed to bridge this gap. These include the DeepCoverMOA and decryptE datasets^6,7^, which represent some of the largest, publicly-available chemical perturbation-based proteomic datasets. DeepCoverMOA analyzed HCT116 cells in response to 875 therapeutic and tool compounds and revealed that a majority of the measurable proteome was accessible to regulation by small molecule treatment. The resulting proteome remodeling responses were then used to assemble proteomic networks to define consistent chemical mechanisms of actions. In a related study, the decryptE dataset studied dose-dependent chemical responses of 144 compounds in Jurkat cells to determine similarities and differences in cellular mechanisms of action. Yet, an important caveat to these two studies was that the whole proteome analysis to decipher compound mechanisms of action were each done in single, isogenic cell lines which may not be representative of the genetic and molecular heterogeneity when considering non-isogenic cell models^8,9^. Interestingly, both the DeepCoverMOA and decryptE datasets evidenced high overall compound activity for histone deacetylase (HDAC) inhibitors (HDACi) such as vorinostat, quisinostat, and nexturastat^6,7^.

HDAC regulation is key to the cellular chromatin state and is frequently dysregulated in cancer^10^ and known to exhibit lineage-specific dependencies in cancer cells^11^. The relationship between HDACs and cancer progression has driven a decades-long effort to develop targeted chemical HDAC inhibitors (HDACi)^12^ and FDA approvals for several of these inhibitors to treat lymphomas and melanomas^13–15^. While HDAC inhibitors have shown promising preclinical results for many cancers, clinical trial results vary^16^. Vorinostat monotherapy in non-small cell lung cancer (NSCLC) patients did not show objective antitumor activity, but toxicity with adverse effects such as fatigue^17^. Other phase II monotherapy of romidepsin and pivaloyloxymethyl butyrate resulted in little efficacy in patients, and had side effects such as fatigue, nausea, and dysgeusia^18,19^.

While a comprehensive understanding of the pleiotropic molecular consequences remains elusive^20^, treatment of cells with HDACi induces expression of p21/CDKN1a leading to cell cycle arrest^21,22^, alters expression of c-Jun^23^ and the apoptotic regulators Bcl-2 and Bcl-xL^24,25^, attenuates AKT/mTOR signaling leading to autophagy^26^, and suppresses IFN-mediated signaling^27^. Recent chemoproteomics analyses have also revealed that heterogeneous responses to HDAC inhibition may be driven in part by the abundance of HDAC protein complex members and the engagement of off-target HDACi binders such as MBLAC2^28^. Thus, these effects have been shown to be both cell-type dependent and cell-type independent, further confounding our understanding of the molecular consequences of HDACi cellular treatments.

Here, we set out to determine the effects of the HDACi treatment on proteome remodeling in non-isogenic cell lines. In particular, we focused on cell treatments with well-established HDACi compounds to build on previous datasets such as DeepCoverMOA and decryptE—including, belinostat, CUDC-101, trichostatin A (TSA), panobinostat, abexinostat, and vorinostat (SAHA). We did this in the context of a genetically diverse panel of lung cancer cell lines with KRAS, EGFR, TP53, CDKN1A, STK11 mutations and integrated proteomic, phosphoproteomic, and transcriptomics to explore drivers of the heterogeneous HDACi responses in these cells. Quantification of histone modifications status in multiple cell lines and thermal stability analyses then allowed us to determine the molecular linkage and effects of on-target and off-target protein engagement with HDAC inhibitors. We have made these data available as a resource (https://github.com/SchweppeLab/HDAC-Perturbation-DataViewer) to understand how chemical perturbation analyses are affected by preclinical cell line choice and exploration of how cellular diversity affects the characterization of small molecule compound mechanisms of action.

## Results

### Cell line selection for HDACi perturbation analyses

We selected five cell lines for the analysis of cell-type-specific proteome profiling. Cell line selection was based on three factors: presence of comparative datasets for benchmarking of the resulting quantitative proteomics analyses, representative mutational status of non-small cell lung cancers (NSCLCs), and divergent responses to epigenetic perturbation the PRISM drug repurposing screen^29^. Based on these criteria, we chose HCT116, A549, PC9, H292, and PSC1 cells as representative cancer cell models to measure the effects of HDACi perturbations.

Two KRAS mutant lines were included—HCT116 (KRAS G13D colon cancer cells) and A549 (KRAS G12S lung adenocarcinoma)—as they have previously been characterized in proteomic and transcriptomic perturbation analyses (Supplementary table 1)^3,6,29,30^. For the remaining cell models, we focused on NSCLC cell lines as this cancer subtype presents divergent preclinical and clinical responses to HDACi treatment^16^. The three additional lung cancer cell lines were chosen for their representative mutational statuses: H292 is a mucoepidermoid carcinoma line (CRTC1-MAML2 fusion, NF2 P496Tfs), PC9 is an adenocarcinoma line (EGFR E746_A750del, TP53 R248Q), and PSC1 is a doxorubicin-resistant derivative of the INER-51 adenocarcinoma cell line (P-gp/MDR-1 negative; Supplementary table 2).

We verified that our selected panel of cell lines was representative of distinct phenotypic responses to small-molecule perturbation. To assess this, we used the PRISM drug repurposing screen, which provides a comprehensive reference of cell viability responses across 911 diverse cell models^2^. We calculated the mean viability fold change for all 911 model cell lines in PRISM. We then compared the global mean of cell viability in response to each drug to the viability for the individual cell lines in our selected panel (A549, PC9, A549, HCT116) as well as the composite mean viability of all four overlapping cell lines. The composite mean of our four cell lines better recapitulated the global PRISM data viability data (four cell lines: r_Pearson_ = 0.836) than any one of our cell lines (PC9: r_Pearson_ = 0.706; HCT116: r_Pearson_ = 0.702; H292: r_Pearson_ = 0.625; A549: r_Pearson_ = 0.593, Supplementary Fig. 1A, Supplementary Data 4).

### Focusing on HDAC inhibition in cancer cell lines

**Fig. 1:**
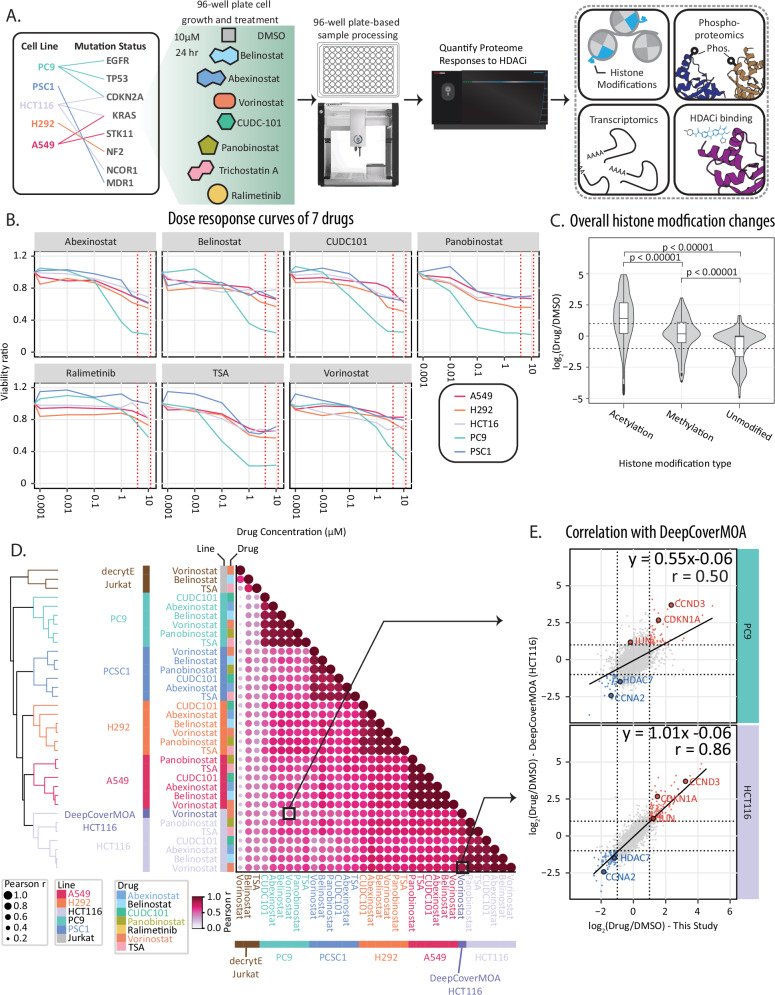
Heterogeneous proteome responses to HDAC inhibition in cancer cells. **A** Overview of the workflow, starting with growing five cell lines in 96-well-plates. Sample preparation was performed with OT2 liquid handler after 24 h drug treatment. The measured Pearson correlation. **B** Dose response curve of each drug tested on 5 cell lines normalized to DMSO treated controls. Viability was measured by luminescent cell viability assay by three biological replicates. Viability ratio is drug treated cells/DMSO treated cells. **C** Violin plot of histone modification abundance changes (center line: median; upper box limit: third quartile; lower box limit: first quartile; whiskers: 1.5x interquartile range; points: outliers). **D** Pearson correlation analysis comparing HDACi treated samples in this dataset (10 μM HDACi, 24 h, HCT116, A549, PSC1, PC9, H292), DeepCoverMOA (10 μM HDACi, 24 h, HCT116), and decryptE (10 μM HDACi, 18 h, Jurkat). **E** Scatterplots highlight specific results from the correlation analysis for (top) the poorly correlated vorinostat-treated HCT116 cells from the DeepCoverMOA study compared to vorinostat-treated PC9 cells from this study and (bottom) highly correlated vorinostat-treated HCT116 cells from the DeepCoverMOA study compared to vorinostat- treated HCT116 cells from this study. The log_2_FC is calculated by comparing drug treatment with DMSO.

Proteomic studies of the responses of cells to small-molecule drug perturbations have been a powerful means to understand diverse mechanisms of action for therapeutic and tool compounds^6,7,31^. To understand how cancer cell lines with non-isogenic backgrounds respond to pleiotropic effects of drug perturbations, we sought to measure proteomic remodeling of multiple cancer cell lines with diverse, and representative, genetic backgrounds in the context of lung cancer—including KRAS, EGFR, TP53, CDKN1A, and STK11 mutations (Fig. 1A). We focused on HDAC inhibition^7,31^, as epigenetic inhibition is a promising target across multiple malignancies and based on the strength of proteome remodeling previously reported in proteomic (decryptE and DeepCoverMOA) and transcriptomic (Sci-plex) datasets^3,6,7^.

We selected six HDAC inhibitors with known canonical and off-target effects, including belinostat (pan-HDACi), CUDC-101 (pan-HDACi, HER2/EGFR inhibitor)^10,32^, trichostatin A (TSA, pan-HDACi), panobinostat, abexinostat (HDAC1/2/3/6/8/10 inhibitor)^33^, and vorinostat (pan-HDACi). To ensure that we could robustly capture proteome remodeling effects at the cell-type- and compound-specific level, we also treated these cell lines with the p38 kinase inhibitor ralimetinib as a non-HDACi-treated outgroup. We performed viability assays and dose response curves to measure the cellular effects of HDAC inhibition (Fig. 1B, Supplementary Fig. 1B, Supplementary Data 3, Supplementary Data 4). We found that a drug concentration of 10 μM generally maintained cell viability relative to DMSO treatment controls of  50% viability or greater after treatment. This was consistent with clinical observation of HDAC inhibitors in patient plasma at levels up to 8 ± 4 μM after treatment^34^.

Owing to the clinical concentrations and collection of previous benchmarking perturbation proteomics datasets, we performed our proteomics analyses at 10 μM of HDACi for 24 h. At this time point, histone acetylation increased with HDAC treatment in H292 and A549 cells and the relative abundance of histone methylation events decreased (Fig. 1C, Supplementary Data 3). As expected, each of the cell lines was differentially sensitive toward the seven tested compounds (Supplementary Fig. 1B). PC9 cells were highly sensitive to HDACi and ralimetinib treatment, especially CUDC-101 (Fig. 1B). The sensitivity to CUDC-101 was likely driven by the known off-target inhibition of EGFR by CUCD-101 as PC9 cells harbor a deletion of E746-A750 in EGFR and EGFR gene amplification ^35^Additionally, A549 cells were sensitive to belinostat to a greater extent than the other HDAC inhibitors. Conversely, PSC1 cells were largely insensitive to HDACi and ralimetinib treatment.

### Proteome quantification of cancer cell responses to HDAC inhibition

Proteome remodeling due to HDACi was measured in cancer cells treated with 10 µM of each compound or vehicle control (DMSO) for 24 h prior to proteomic and viability assays. In total, we performed quantitative proteomic analysis of the 35 single-perturbation, cell-by-drug combinatorial treatments (Fig. 1A). We quantified the relative abundance of proteins in comparison of vehicle (DMSO) controls for each of these perturbations and replicate analyses generated highly correlated proteome abundance shifts (Fig. 1A). In total, we acquired quantitative measurements for 476,387 peptides, 6,611 phosphorylation sites, 177 histone sites, and 10,237 unique proteins. Protein quantification between biological replicates was highly reproducible with a median Pearson correlation (r) for biological replicates of each drug and cell line of 0.996 (Supplementary Fig. 1C). The median coefficient of variation for protein measurements between biological replicates was 4.4% (Supplementary Fig. 1D).

Of 10,237 total proteins we quantified, 6,145 proteins were found in all five cell lines (Supplementary Fig. 1E, Supplementary Data 4), 8,083 proteins were found in at least three cell lines, and 1,311 were found in only one cell line (Supplementary Fig. 1F, Supplementary Data 4). The six HDACi and ralimetinib all have the potential to drive pleiotropic remodeling of proteins and protein complexes^16,36^. Interestingly, though the HDACi treatment drove strong proteome remodeling, PCA analysis clustered cell-line-by-drug groups based predominantly on cell lines, not the compound used to treat these cells. The one notable difference was that compared to matched vehicle controls in each cell line, ralimetinib-treated cells clustered together in principal component space irrespective of the cell line (Supplementary Fig. 1G, Supplementary Data 4).

### Comparison of cell-type specific proteome responses to benchmarking datasets

Comparison of our proteomic data to the DeepCoverMOA dataset in HCT116 cells^6^ and decryptE dataset in Jurkat cells^31^ allowed us to compare consistent and inconsistent responses to HDACi treatment in non-isogeneic cellular backgrounds (Fig. 1D, Supplementary Data 3). Proteome remodeling of HCT116 cells treated with vorinostat in our dataset were highly correlated with HCT116 cells treated with vorinostat in DeepCoverMOA (r_Pearson_ = 0.86, slope = 1.01, Fig. 1E). Correlation between vorinostat-treated HCT116 cells in DeepCoverMOA and vorinostat treatments in our data was lower (r_Pearson_ = 0.50–0.69, Fig. 1D).Consistent with DeepCoverMOA and decryptE, the abundance of CDKN1A, CCND3 and c-Jun increased with HDACi treatment^21–23^, while the abundance of CCNA2 decreased with HDACi treatment^21,22^ (Fig. 1E).

A549 cells harbor similar oncogenic KRAS and SMARCA4 mutations to HCT116 cells. When treated with vorinostat, A549 cells also had the highest correlation to HCT116 cells in the DeepCoverMOA vorinostat treatment (r_Pearson_ = 0.69). PC9 cells with vorinostat had the lowest correlation compared to the DeepCoverMOA data (r_Pearson_ = 0.50, Fig. 1E). PC9 cells were also the most sensitive to HDACi perturbations (Fig. 1B) and they did not have the increased abundance of JUN due to HDACi as seen in the other four cell lines (Fig. 1E). Comparing our belinostat, TSA, and vorinostat treatment data and the decryptE analysis of HDACi treatment of Jurkat cells^31^, we observed lower overall correlation with the decryptE Jurkat cells responses to HDACi (r_Pearson_ = 0.15–0.50, Fig. 1D). These data highlight the importance of considerations of the cellular context of cells when assessing mechanism of action and polypharmacology using proteomics.

### HDAC inhibition drives cell-type specific alteration of HDAC but not histone protein abundance landscape

**Fig. 2:**
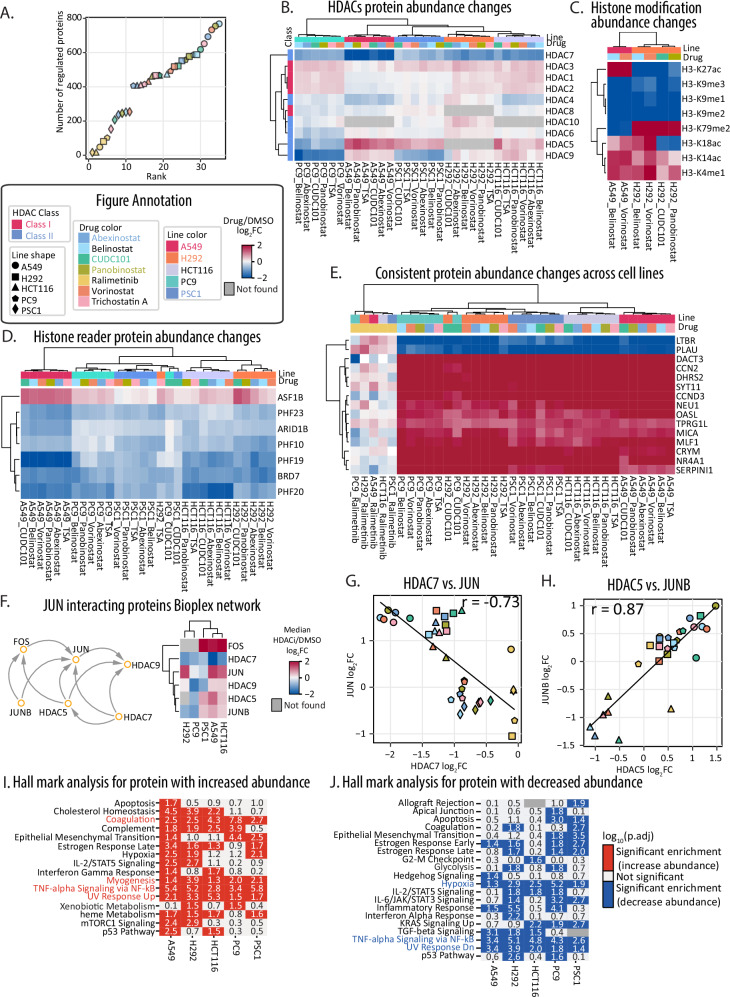
Response of HDAC and HDAC-related proteins to HDAC inhibition. **A** Waterfall plot highlighting the number of regulated proteins for each cell-line-by-drug group. **B** Heatmap of protein abundance changes of 10 HDACs in 5 cell lines with HDACi treatment. **C** Heatmap of histone modification abundance changes regulated in A549 and H292 with HDACi treatments. **D** Heatmap of protein abundance changes of histone binding proteins in 5 cell lines with drug treatments. **E** Heatmap of protein abundance changes of 15 proteins having abundance changes in all 5 cell lines with all HDACi treatments. **F** Protein network from Bioplex between HDAC5/7/9 and JUN. The heatmap represents median protein abundance changes of HDAC5/7/9 and JUN with HDACi treatment in 5 cell lines. **G** Comparison of protein abundance changes between HDAC7 and JUN. **H** Comparison of protein abundance changes between HDAC5 and JUN. Pearson r is labeled on the top of the figure. **I**, **J** Comparison of Hallmark enrichment analysis of regulated proteins from cell lines with HDACi treatment. Left with red color (**I**) is comparison of up-regulated proteins; right with blue color (**J**) is comparison of down-regulated proteins. Regulated proteins are proteins with absolute log_2_FC > 1. The p-value is calculated by comparing the observed frequency of an annotation term with the frequency that would be expected by chance. The p-value is adjusted by the Benjamini-Hochberg false discovery rate (FDR). Terms that have an adjusted *p*-value (p.adj) < 0.05 or log_10_(p.adj) > 1.3 are considered enriched. Both color scale and numbers represent log_10_(p.adj). For all figures above, the log_2_FC is calculated by comparing drug treatment with DMSO.

In total, we observed 16,713 cell-line-by-drug regulated events across all groups on 2553 different proteins (|log2FC | > 1, Supplementary Fig. 2A, Supplementary Data 1, Supplementary Data 4). Among the 2553 regulated proteins, we quantified 2282 proteins that were differentially abundant in less than 14 cell-line-by-drug groups—suggesting cell type- or cell state-specific responses (Supplementary Fig. 2A, Supplementary Data 3). Based on the total number of regulated proteins, we observed the largest degree of proteome remodeling in HDACi-treated A549 cells (Fig. 2A, Supplementary Fig. 2B). Conversely, our outgroup treatment of cells with ralimetinib generated the lowest number of regulated proteins (Fig. 2A). Chemoresistant PSC1 cells had the lowest number of regulated proteins among all tested cell lines which is consistent with the limited effect of drug treatment on cellular viability in these cells (Fig. 2A, Supplementary Fig. 2B). When comparing compound-specific effects, belinostat treatment of lung cancer cells (A549, H292, PC9 and PSC1) consistently generated the largest degree of proteome remodeling, followed by panobinostat, abexinostat, TSA, CUDC-101 and vorinostat (Fig. 2A).

Across all five cell lines, we quantified 10 of the 11 human HDAC proteins in response to HDACi (Fig. 2B, Supplementary Data 3). HDAC11 was not observed likely due to its generally low abundance in lung cells^37^. We observed changes in both individual HDAC abundances and more generally in the responses of classes of HDACs. Class I HDACs include HDAC1, 2, 3, and 8, and these are generally found in the nucleus^10^. Class II HDACs include HDAC4, 5, 6, 7, 9, and 10, which shuttle between the nucleus and cytoplasm^10^. In our analysis, the abundance of Class I HDACs generally did not change after HDACi treatment, but we observed differential abundance for three out of six Class II HDACs (HDAC5/7/9, Fig. 2B). HDAC7 protein abundance decreased across all cell lines, HDAC9 protein abundance decreased in PC9 and PSC1, while HDAC5 protein abundance increased in A549, PSC1 and HCT116, but decreased in PC9 (Fig. 2B). Previous small-scale analyses found that HDAC7 (Class II), but not HDAC1 (Class I), protein abundance decreased with HDACi treatment^38,39^.

While HDAC protein abundances differed by class, the relative abundance of most histones did not have significant changes with HDACi treatment (Supplementary Fig. 2C, Supplementary Data 4). Yet, we did find that histone modification status had cell line-specific HDACi-mediated responses (Fig. 2C, Supplementary Data 3). Comparing A549 and H292 cells, the majority of histone modifications were consistent, e.g., the abundance of H3K14ac, H3K18ac and H3K4me1 increased with HDACi treatment, and the abundance of H3K9me1, H3K9me2 and H3K9me3 decreased with HDACi treatment (Fig. 2C). Conversely, H3K27ac abundance increased as expected in A549 cells treated with HDACi but decreased in H292 cells. Similarly, H3K79me2 had increased abundance in H292 cells upon HDACi treatment, but reduced abundance in A549 cells (Fig. 2C). Indeed, H3K79 methylation can antagonize modification of H3K27^40^. We note that the protein abundances of histone methyltransferases did not change in either cell line—e.g., for SETD2 which is associated with H3K36 methylation^41^ or EZH1/EZH2 which are associated with H3K27 methylation^42^.

### Cell-type-specific regulation of histone modifications and chromatin regulatory factors with HDAC inhibition

In addition to HDAC and histone abundance, we investigated whether known histone binding proteins had differential protein abundance with HDACi treatment. Seven histone readers had abundance changes with HDACi treatment (Fig. 2D, Supplementary Data 3), including histone chaperone ASF1B (histone H3 binder), histone reader BRD7 (Histone H3 binder), PHD finger proteins (PHF10/19/20/23), SWI/SNF chromatin remodeling protein ARID1B^43–47^.

Among the 7 histone readers, ASF1B, BRD7, and PHF10 bind H3K14ac^44,45,48^. The histone chaperone ASF1B recognizes H3K9me1 and imports H3 into the nucleus, where ASF1B can form a complex with H3K14K18ac^44^. H3K9me1 abundance decreased in A549 cells treated with belinostat or vorinostat treatment, while H3K14ac and H3K18ac increased (Fig. 2C, Supplementary Fig. 2D, Supplementary Data 4). The abundance of PHF10 and BRD7 decreased with HDACi treatment, which was inverted from the phenotype we observed for ASF1B (Fig. 2D). HDACi treatment resulted in increased H3K27ac in A549 cells, but decreased H3K27ac in H292 cells (Fig. 2C, Supplementary Fig. 2D). ASF1B overexpression is known to promote proliferation of cancer cells in a CDK9-dependent manner^49^. Yet, we observed no significant change in CDK9 protein abundances (Supplementary Data 1). These data hint at a CDK9-independent route by which ASF1B stabilization in cancer cells may play a role in bypassing the effects of HDAC inhibition.

### Altered response of HDAC pathway members with HDACi treatment

Among the 2553 proteins with significant HDACi-induced abundance changes, we found only 15 proteins with significantly altered abundances in all HDACi-treated cells (Fig. 2E, Supplementary Data 3). We found this to be a striking result, but consistent with the poor correlation we observed with some previous datasets when comparing proteome responses after HDACi treatment (Fig. 1E, Supplementary Data 3). The 15 proteins that changed consistently in all cell lines included known HDACi responsive proteins, such as increased protein abundance of the Wnt-signaling related protein DACT3^50^ and the cell cycle regulatory protein cyclin-D3 (CCND3)^51,52^. More broadly, these 15 proteins were enriched for Wnt signaling and lung fibrosis annotations (Supplementary Fig. 2E). We also found that several proteins previously annotated as HDACi responsive had significant abundance changes in a subset of our tested cell lines. These included increased abundance of cyclin-dependent kinase inhibitor 1 (CDKN1A)^51,52^; increased abundance of transcription factor and oncoprotein c-Jun (JUN)^39^; decreased abundance of HDAC7^39^; and reduced abundance of cyclin-A2 (CCNA2)^53^.

Based on these HDAC-class-specific changes, we sought to determine how HDACi treatment remodeled the canonical HDAC regulatory pathway components, such as the JUN signaling pathway. To explore the proteomic relationships surrounding JUN and HDACi treatments, we expanded the analysis to include direct interacting proteins of JUN based on the BioPlex interaction network^54,55^ (Fig. 2F, Supplementary Data 3). Within the JUN interaction network, the c-Jun transcription factor (JUN) had a significant increase in protein abundance in A549, H292, and HCT116 cells (log_2_FC_A549_ = 1.49; log_2_FC_H292_ = 1.43; log_2_FC_HCT116_ = 1.20), but not PC9 and PSC1 cells (log_2_FC_PC9_ = −0.09; log_2_FC_PSC1_ = −0.37; Fig. 2F). We observed distinct differences in the abundance of the transcription factor JunB which decreased in H292 and PC9 cells (log_2_FC_H292_ = −0.84; log_2_FC_PC9_ = −1.1), but not in A549, HCT116 and PSC1 cells (log_2_FC_A549_ = 0.62; log_2_FC_HCT116_ = 0.30; log_2_FC_PSC1_ = 0.40, Fig. 2F). We observed the same trend for c-FOS which forms a heterodimer with JUN and JunB, as c-FOS protein abundance significantly increased in A549, HCT116 and PSC1 cells (log_2_FC_A549_ = 1.82; log_2_FC_HCT116_ = 2.5; log_2_FC_PSC1_ = 3.62, Fig. 2F).

We observed a negative correlation between JUN abundance and HDAC7 abundance and a positive correlation between JUNB and HDAC5 abundance (Fig. 2G, H, Supplementary Data 3). However, there is no significant correlation between HDAC9 and JUN or JUNB. In the case of HDAC7, HDACi treatment induced decreased abundance of HDAC7 and increased abundance of JUN (Fig. 2G). Since HDAC7 can inhibit JUN expression^39^, these proteomic profiles suggest that decreased abundance, and thereby activity, of HDAC7 may be driving JUN abundance in response to HDACi. Moreover, the HDAC7 and JUN relationship was specific to HDACi-perturbed cells as no significant relationship between JUN and HDAC7 protein was observed in the large-scale CCLE proteomics screen of unperturbed cell lines^30^, (Supplementary Fig. 2F, Supplementary Data 4). HDACi treatment induced increased abundance of both HDAC5 and JUNB, a phenotype similar to that seen in which is similar to the injury-induced HDAC5 activity regulation of JUNB expression^56^.

Beyond the relationship of JUN/JUNB with HDACi treatment, enrichment analysis^57^ found that HDACi-responding proteins were significantly enriched for TNF-alpha signaling via NF-kB and UV response pathways (Fig. 2I, J, Supplementary Data 5). Additional pathways were only significantly regulated in a specific subset of cell lines. For example, mTORC1-associated proteins were onsly enriched in A549 and H292 cells treated with HDACi. Notably, this did not correlate with the background mutational status of these cell lines as A549 cells are driven by a constitutively-active KRAS mutant and H292 cells are driven by the loss of the NF2 tumor suppressor^58^. Interestingly, proteins involved in p53 pathways mainly had increased abundance in A549 and HCT116 cells, while they had decreased abundance in H292 and PC9 cells, and these proteins were not differentially regulated in PSC1 cells.

### Identifying direct effects of HDACi treatment on the proteome

**Fig. 3:**
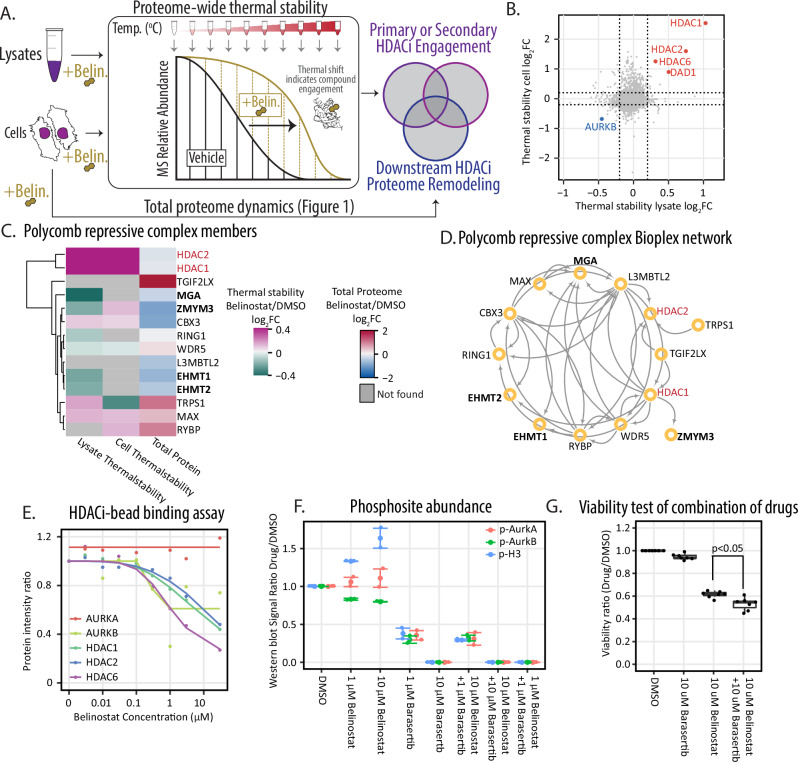
Analysis for protein-drug engagement for divergent cellular responses. **A** Diagram shows cell and lysate thermal stability analyses and total proteome were performed using A549 cells or lysates treated with 10 μM belinostat. **B** Comparison between two thermal stabilities of A549 with belinostat treatment. The log_2_FC is calculated by comparing drug treatment with DMSO. **C** Heatmap of protein abundance changes in two thermal stability assay and total proteome responses of PRC1 members. **D** Network from Bioplex of components of the polycomb repressive complex (PRC1). **E** Dose-dependent response curve of AURKB to HDACi treatment from HDAC-targeting pharmacophores (Lechner et al. 2022). **F** Western blot signal measured with ImageJ of AURKB, AURKA/B autophosphorylation and H3 Ser10 phosphorylation with belinostat and barasertib treatment. Phospho-signals were normalized with the AURKB protein signal. Drug treatment signal was compared with DMSO treatment signal Western blot signal measurements were measured three times. Error bars represent standard deviation. **G** Viability assay of A549 treated with combination of belinostat and barasertib. *N* = 7 biological replicates.

After 24 hours of treatment with HDACi, the protein abundances for HDAC1 and HDAC2 did not change (Fig. 2B), suggesting additional mediators of HDACi responses. To identify these mediators, we employed thermal proteome profiling to measure direct effects of HDACi treatment of A549 cells^59,60^ (Fig. 3A). Owing to the potential importance of protein, cellular, and chromatin context in affecting HDACi engagement, we performed these assays in both cells and lysates to confirm protein-engagement data and determine likely direct versus indirect HDACi relationships^60^. In the presence of belinostat, HDAC1, HDAC2 and HDAC6, HDAC2 were significantly stabilized (Fig. 3B, Supplementary Data 3). We also observed HDACi-mediated destabilization of HDAC1/2 containing complex components, including polycomb repressive complex components MGA and EHMT1/2 (Fig. 3C, D, Supplementary Data 3)^61^, and the BHC histone deacetylase complex component ZMYM3^62,63^.

In addition to thermal stability shifts for HDACs and HDAC-related proteins, HDACi altered the thermal stability of potential off-target proteins, including the cell cycle kinase AURKB (Fig. 3B). AURKB was significantly destabilized with HDACi treatment in both cell- and lysate-based thermal stability analyses (Fig. 3B). Re-analysis of bead-based small-molecule pulldown assays using HDAC-targeting pharmacophores^28^ revealed a dose-dependent response of AURKB to HDACi treatment similar to HDAC1/2/6, but not dose dependent response for the structurally similar AURKA (Fig. 3E, Supplementary Data 3).

Measurement of A549 cellular responses confirmed that belinostat treatment increased AURKB-dependent phosphorylation of H3 Ser10 (Fig. 3F, Supplementary Data 3, Supplementary Fig. 3A, Supplementary Fig. 6A–C). Addition of the AURKB inhibitor barasertib treatment, abolished H3 Ser10 phosphorylation. Consistently, phosphorylation on AURKB substrates and interacting partners INCENP and RACGAP1 increased with belinostat treatment (Supplementary Fig. 3B, Supplementary data 4). Based on these findings, we tested whether combination treatment of HDACi and AURKB inhibitors would further inhibit cell viability. Indeed, the combination of belinostat and barasertib treatment significantly reduced cell survival compared to either belinostat or barasertib alone (Fig. 3G, Supplementary Data 3).

### Co-regulatory analyses reveal a linkage between HDAC7 abundance and 14-3-3 protein interactions

**Fig. 4:**
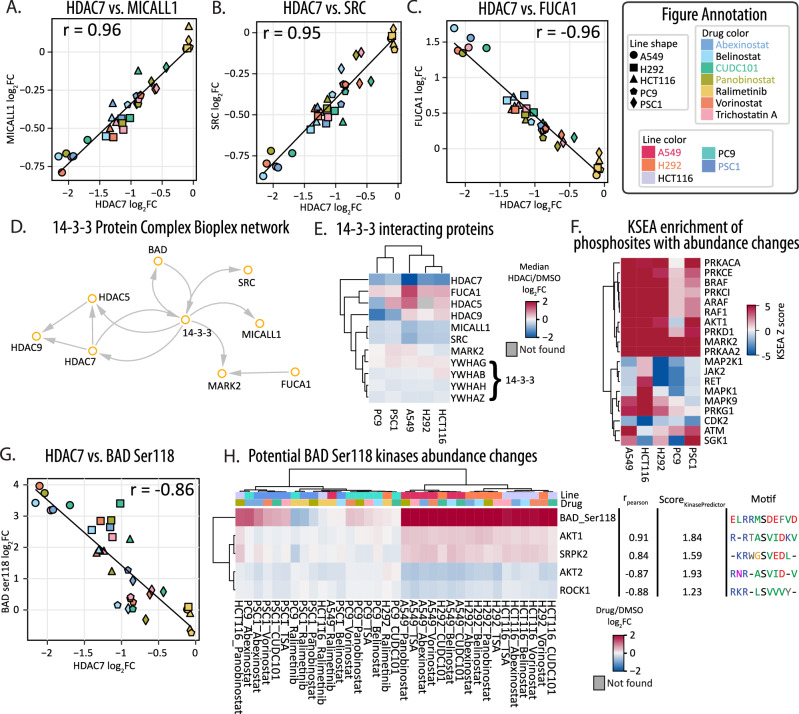
Altered response of 14-3-3-related proteins and phosphorylation. **A–C** Comparison of protein abundance changes between HDAC7 and MICALL1 (**A**), SRC (**B**) or FUCA1 (**C**). Pearson r is labeled on the top of the figure. **D**, Network from Bioplex between 14-3-3 adapter proteins with HDAC5/7/9, MICALL1, SRC and BAD. **E** Heatmap of median protein abundance changes of HDAC5/7/9 and 14-3-3 related proteins with HDACi treatment in 5 cell lines. **F** KSEA enrichment of phosphosites having abundance changes with HDACi treatment in 5 cell lines. **G** Comparison of abundance changes between HDAC7 and BAD ser118 phosphorylation. Pearson r is labeled on the top of the figure. **H** Heatmap of phosphorylation abundance changes of BAD ser118 and protein abundance changes of correlated kinases. For all figures above, the log_2_FC is calculated by comparing drug treatment with DMSO.

In addition to direct effectors of heterogeneous HDACi response, we explored whether specific sets of proteins or protein complexes were co-regulated with HDACi treatment. Correlation analysis was performed comparing the 10 quantified HDACs and the 5,491 proteins quantified in all cell line-by-drug samples (Supplementary Data 6). HDAC1 and HDAC2 are known to exhibit partially redundant effects in cells^11^, and we observed that HDAC1 and HDAC2 protein abundances were correlated across datasets (r_Pearson_ = 0.92). Using this correlation approach, we observed 81 proteins with correlated protein abundances to at least one HDAC protein (r_Pearson_ > 0.9, Supplementary Fig. 4A, Supplementary Data 4). In keeping with the cell line specific response of HDAC7 to HDACi treatment, we observed 83.9% of these proteins (68 of 81) were correlated with HDAC7 abundance (Supplementary Fig. 4A). Of these proteins, the most highly correlated proteins (r_Pearson_ > 0.95) with HDAC7 were the tyrosine kinase SRC, MICALL1, and the fucosidase FUCA1 (Fig. 4A–C, Supplementary Data 3).

The tyrosine kinase SRC is active in non-small cell lung cancers and involved in cellular transformation and metastasis^64^. Yet, consistent with our proteomics analysis, HDACi treatment can reduce SRC expression in cancer cells^65^. While there was limited functional information about the role of MICALL1 (Molecule Interacting with CasL-like 1) in cancer, MICALL1 protein abundance in response to HDACi treatment was highly correlated with both HDAC7 (r_Pearson_ = 0.96) and SRC (r_Pearson_ = 0.90) protein abundance. In steady state protein abundance measurements across CCLE cancer cells, SRC, MICALL1, and FUCA1 had weak anticorrelation with HDAC7 (Supplementary Fig. 4B–D, Supplementary Data 4). Thus, HDACi treatment seems to drive the coordinated protein abundances of these proteins potentially through shared interactions with 14-3-3 adapter proteins (YWHAH, YWHAG, YWHAB, YWHAZ, Fig. 4D, E, Supplementary Data 3)^54,55,66–69^. The 14-3-3 proteins are known to regulate the nuclear import of Class IIa HDACs (HDAC4/5/7/9) and bind phosphorylated HDAC7^70,71^. Though many of the HDACi compounds used in our study primarily target both Class I and II HDACs^10^, we found that the Class II HDAC7 exhibited robust, but cell-type-specific, responses to HDACi treatment across multiple lung cancer cells lines.

### HDACi alters phospho-signaling surrounding 14-3-3 regulated proteins

Based on the relationship between 14-3-3 proteins’ binding of phosphosites and their regulation of HDACi responses, we performed phosphoproteomics on HDACi treated cells. We identified a total of 6611 phosphosites, including 2271 phosphosites on 1244 proteins with HDACi-dependent abundance changes (Supplementary Data 2). Based on Kinase-Substrate Enrichment Analysis (KSEA)^72^ of phosphosites with HDACi-dependent abundance changes, activities for MARK2 and PRKAA2 were enriched in all cell lines. Activities for 8 additional kinases were enriched in all cell lines except PC9, and 9 kinase activities were enriched in at least one cell line (Fig. 4F). PC9 cells treated with HDACi had the largest number of regulated sites, followed by H292, A549, HCT116, and PSC1 cells (Supplementary Fig. 4E, Supplementary Data 4). PC9 cells were also the most susceptible to HDACi treatments in cell viability assays (Fig. 1A). Cell lines treated with the p38 kinase inhibitor ralimetinib had the fewest regulated phosphosites (Supplementary Fig. 4E).

The protein abundances of class II HDACs after HDACi treatment were highly correlated or anticorrelated with 27 phosphosites: HDAC5 (n_sites_= 12), HDAC7 (n_sites_= 11), and HDAC9 (n_sites_= 4) (r_Pearson_ > 0.8, quantified in at least 80% of our cell-line-by-drug groups, Supplementary Fig. 4F, Supplementary Data 4). Related to the differential viability of cells in response to HDACi, the regulatory phosphosite Ser118 on the cell death regulator BAD was anticorrelated with HDAC7 abundance (r_Pearson_ = −0.86, Fig. 4G). Phosphorylation of BAD Ser118 disrupts Bcl-2/Bcl-XL binding inhibiting BAD-mediated apoptosis^73,74^. BAD Ser118 phosphorylation increased with HDACi in the three cell lines with intermediate sensitivity to HDACi (A549, H292, HCT116; Figs. 1A, 4G). Conversely, no significant co-regulation was observed between HDAC7 and BAD protein abundance (r_Pearson_ = 0.13). BAD Ser99 phosphorylation, generally thought to act upstream of Ser118 phosphorylation, was also unchanged in response to belinostat (Supplementary Data 2). Analysis of the decryptM phosphoproteomics responses confirmed vorinostat and abexinostat treatments increased BAD Ser118 phosphorylation with no change to Ser99 phosphorylation in MV4-11 and HeLa cells^7^. These data point to a cell-type-specific relationship between HDAC activity (and HDACi inhibition), HDAC abundance, and a BAD-dependent mechanism to overcome HDACi-mediated cell death.

Based on the consistent cell-type-specific responses, we sought to determine which kinases may be responsible for the HDACi-dependent phosphorylation of BAD. Previous work demonstrated that Ser118 can be phosphorylated by multiple kinases, including RAF, PKA, RSK, and AKT1^74–76^. To determine which kinases may be responsible for Bad Ser118 phosphorylation, we performed coregulation analysis^6^ between Ser118 abundance and the protein abundances of 248 protein kinases quantified in at least 28 HDACi-treated samples. In addition, based on the ‘RR-x-S’ motif surrounding BAD Ser118, we ran KinasePredictor^77^. Among 77 kinases found in our data, AKT1 abundance changes had strong correlation with BAD Ser118 abundance changes (r_Pearson_ = 0.91), as well as a high KinasePredictor score (1.83). In addition to AKT1, there are three more kinases that have KinasePredictor score larger than 1 and protein abundance changes were correlated with BAD Ser118 abundance (absolute r_Pearson_ > 0.8). These included AKT2 (r_Pearson_ = −0.87, Score_KinasePredictor_ = 1.93), ROCK1 (r_Pearson_ = −0.88, Score_KinasePredictor_ = 1.23) and SRPK2 (r_Pearson_ = 0.84, Score_KinasePredictor_ = 1.59) (Fig. 4H, Supplementary Data 3).

### Coordinated remodeling of the transcriptomes and proteomes of HDACi perturbed cells

**Fig. 5:**
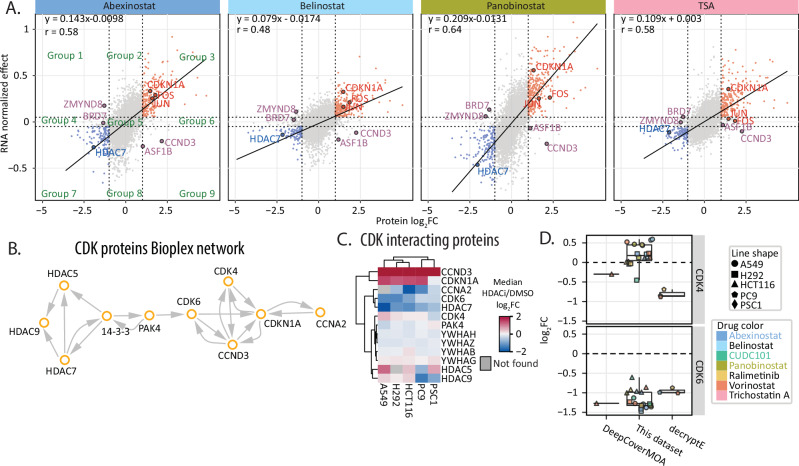
Coordinated transcriptome and proteome responses in A549 with HDACi treatments. **A** Activity of A549 treated with 4 HDACi by the protein abundance log2FC (x-axis) compared with DMSO and RNA normalized effect (y-axis). The regulated proteins were defined as absolute log_2_FC > 1 in the proteomics dataset and absolute normalized effect > 0.05 in transcriptome dataset. Based on the four thresholds, proteins were grouped into 9 clusters. **B** Network from Bioplex between 14-3-3 adapter proteins with HDAC5/7/9 and cyclin-dependent kinases. **C** Heatmap of median protein abundance changes of 14-3-3 adapter proteins, HDAC5/7/9, and cyclin-dependent kinases with HDACi treatment in 5 cell lines. **D** Boxplot of protein abundance changes of CDK4 and CDK6 in this dataset, DeepCoverMOA and decryptE with HDACi treatments. For all figures above, the log_2_FC is calculated by comparing drug treatment with DMSO.

Owing to the inherent relationship between HDAC activity and transcription, we compared A549 drug-perturbed proteomics data to pseudo-bulked single-cell transcriptome with matched treatment conditions (abexinostat, panobinostat, belinostat, TSA)^3^, Supplementary Fig. 5A). There were 232 transcripts with shared differential expressions between proteins and transcripts in all 4 treatments (Supplementary Fig. 5B). In response to HDACi, A549 cellular proteomes and transcriptomes were generally correlated (r_Pearson_ = 0.48–0.64, Fig. 5A, Supplementary Data 3), consistent with previous work (r_Pearson_ = 0.42–0.57)^5^. We grouped protein and transcript responses to HDACi treatment into nine groups based on significant changes in proteomic and transcriptomic responses—e.g., Group 1 had increased transcript expression decreased protein abundance with HDACi treatment while Group 3 had concordant increased transcript expression and increased protein abundance with HDACi treatment (Supplementary Fig. 5C).

Concordant transcript and protein responses to HDACi treatment in Groups 3 and 7 were observed for 505 protein/transcript pairs. As expected, the proteins in these groups were enriched for oncogenes and tumor suppressors, with notable effects of increased abundance of JUN, FOS and CDKN1A, and reduced abundance of HDAC7 and CCNA2 (Fig. 5A). We then defined protein- or transcript-specific responders in Groups 1, 4, 6, and 9—i.e., those with differential abundance of proteins with HDACi treatment, and no effect on the transcripts, or vice versa (Fig. 5A). With HDACi treatments, cyclin D3 (CCND3) was discordantly regulated with increased protein abundance after HDACi treatment and decreased transcript expression (Fig. 5A). Conversely, the cyclin-dependent kinase inhibitor CDKN1A had a concordant relationship between its transcript and protein abundances with HDACi treatments (Fig. 5A).

Since CCND3 coordinates cell cycle progression through binding and activation of CDK4 and CDK6 kinases, the discordant proteomics and transcriptomic profiles hint at a role of post-translational control of CCND3 protein abundance. One route for post-translational control of CCND3 could be through stabilization of the CDK4-CCND3 or CDK6-CCND3 protein complexes^78–80^ (Fig. 5B). We found that CDK6, but not CDK4, protein abundance decreased in A549 cells with HDACi treatment (Fig. 5C, Supplementary Data 3). Consistent with DeepCoverMOA and decryptE data, s, we found that CDK6 protein abundance significantly decreased with HDACi treatment in the DeepCoverMOA and decrytE datasets (Fig. 5D, Supplementary Data 3)^6,31^. However, CDK4 protein abundance was not significantly changed in our data or in DeepCoverMOA’s HCT116 study, but CDK4 abundance did decrease in Jurkat cells suggesting a route for heterogenous cell cycle responses to HDACi treatment (decryptE, Fig. 5D).

## Discussion

Disparate cell states within cancer cells, driven by differential genetics, signaling axis activation, and protein interactions lie at the heart of inconsistent outcomes for therapeutic treatments. We investigated this phenomenon in the context of pleiotropic epigenetic (HDAC) inhibition as a model. HDAC inhibitors are actively used clinically for hematological malignancies and also promising preclinical activities for solid tumors, but with disputed efficacy based on cancer type and known adverse effects^16^. Our multiomic study reinforces that it is essential to characterize the molecular outcomes of therapeutic intervention that represent the diversity of molecular starting points (e.g., genetic backgrounds). In the context of lung cancer cell models, the majority of cell line models have a remarkable imbalance of race and gender, which calls for action to establish omics resources to characterize these heterogeneous responses to drugs^81^. Our work delineates the heterogeneity of proteome remodeling with drug treatment, was consistent with heterogeneity in the cell line viability analysis and can offer important clues as to the differential susceptibility of these cell models to HDACi perturbation.

At the outset of this work, several large databases existed for the characterization of diverse in human cancer cell lines, such as recent chemical genomic^3^ and proteogenomic characterization of CCLE cell lines^30,82^. Here, we focused on measuring steady-state proteome remodeling of genomically diverse cancer cell lines after exposure to drug perturbation. We compared our lung cancer-specific proteomics data with recent databases describing mechanistic annotation of drugs by profiling proteomics data, such as DeepCoverMOA^6^, and the decrypt datasets^7,31^. In shared cell-by-drug sample sets with these datasets, we observed consistent HDACi perturbation effects on the abundance of CDKN1A and CCND3^51,52^; increased abundance of JUN protein and decreased abundance of HDAC7^39^; and reduced abundance of CCNA2^53^.

Our data from multiple cell lines also revealed new heterogeneity in HDACi treatment response. The protein abundance of JUN in A549, H292 and HCT116 cells, but not regulated in PC9 cells, correlated with PC9 cells’ poor viability with HDACi treatment. Upregulation of JUN is associated with cell proliferation and differentiation^83^, which could explain PC9 is more sensitive to HDACi. Second, owing to the use of a single cancer cell line in many of these studies^6,31^, our data added a new level of understanding for how non-isogenic cell lines respond to chemical perturbation. Building on this work, the data presented here provides a molecular landscape for how cancer cell lines behave when they are treated with epigenetic inhibitors, and how this information can be leveraged for therapeutic treatments.

We built a web interface for the extensive amount of data to allow users to explore the data as a resource for understanding chemical perturbation of diverse cell types (https://github.com/SchweppeLab/HDAC-Perturbation-DataViewer). We explored the non-negligible differences in drug responses across various lung cancer cell lines through proteome profiling of five distinct lines by integrating proteomics, phosphoproteomics, and histone modification data. By combining proteomics with thermal stability data and transcript profiles, we identified specific protein and protein complex effectors of HDACi treatment in lung cancer cells (e.g., polycomb variant PCGF6). HDACi engagement data also revealed potential mechanisms of HDACi, such as through the engagement of DAD1 and AURKB. From our viability assay, A549 cells were more sensitive to belinostat than any other HDACi and this compound-specific sensitivity was not seen for the other KRAS mutant cell line (HCT116) or the other moderately sensitive H292 cells. Steady state AURKB protein abundance was 5.7-fold lower in A549 cells compared to H292 cells^30^ potentially making A549 cells especially sensitive to off-target inhibition of AURKB.

Discordant changes of transcript and protein abundance revealed multiple potential routes by which cells can compensate for HDACi-initiated cell death. For the histone reader ASF1B that can interact with H3K27ac and H3K79me2^84^, we observed discordant transcript and protein abundance with HDACi treatment. Since the transcript abundance of ASF1B did not change significantly while protein abundance increased, we speculate that ASF1B protein abundance may be regulated post-translationally, potentially through hyperacetylation of H3 leading to nuclear sequestration and protection from degradation. In addition to the potential H3/ASF1B response to HDACi, our data show that abundance changes for specific histone modifications were correlated with protein and/or transcript abundance for cell cycle and chromatin regulators CCND3, ASF1B, PHF10 and BRD7. Since ASF1B, PHF10 and BRD7 can recognize H3K14ac. For BRD7 we note that only through the comparative transcriptomic and proteomic analysis did this protein become a strong candidate for future work owing to the moderate decrease in protein abundance but a discordant increased transcript abundance.

Finally, cell-line models are inherently reductionist and lack microenvironmental, immune, and pharmacokinetic contexts. Therefore, drug responses will not fully represent in-vivo behavior. Given this limitation, by profiling multiple genetically diverse lung cancer lines using proteomics, phosphoproteomics, and histone mapping we were still able to reveal reproducible HDACi signatures. Together, the integration of our HDACi engagement data with previous data enabled identification of proteomic effectors of heterogenous cellular responses to HDACi. Thus, although this dataset focuses on HDAC inhibition, extension of our multiomics approach to include additional epigenetic drugs, larger cohorts of diverse cell lines, and improved methods to enhance throughput would further empower our understanding of key effectors of heterogeneous molecular responses in cell models.

## Methods

### Cell culture conditions

A549 (CVCL_0023), H292 (CVCL_0455), HCT116 (CVCL_0291), PC9 (CVCL_B260), and PSC1 (CVCL_5622) cells were dispensed into tissue culture treated 96 well plates at a density of 37,500 cells per well in a volume of 200 μl. After 24 h, cells growing at less than 80% confluence were then treated with 10 μM of each drug, including abexinostat, belinostat, CUDC-101, trichostatin A (TSA), panobinostat, vorinostat (SAHA), and ralimetinib, or 0.1% DMSO for 24 h. All treatments were performed in 6 replicates: 2 replicates for proteome analysis, 2 replicates for viability analysis and 2 replicates for BCA assay. After treatment, cells were incubated for 24 h at 37 °C before washing with 200 μl PBS per well. After all PBS was aspirated, clear cell culture plates were immediately used for sample preparation for proteomics analysis or viability assay. Viability assay was performed with CellTiter-Glo® Luminescent Cell Viability Assay (Promega).

### Sample preparation for proteomics analysis

As previously described in ref. ^85^, sample lysis, reduction, alkylation, SP3 and digestion were processed with an OT2 liquid handler. Lysis buffer for BCA assay consisted of 8 M urea, 50 mM NaCl, 200 mM EPPS pH 8.5, and Roche protease inhibitor tablets. For reduction, 5 mM TCEP was added to the lysis buffer. Lysis buffer 30 μl was added into each well, followed by shaking at 500 rpm for 30 min. Protein concentrations were measured using Pierce BCA assay kits. Proteins for proteomics analysis were alkylated with 30 μl of 10 mM iodoacetamide for 30 min in the dark. The alkylation reaction was quenched by adding an 5 mM DTT. Proteins were then transferred to a 96-well PCR plate and isolated using SP3. In brief, 4 μl of SP3 bead mix was added to each well before adding 65 μl neat ethanol and shaking for 30 min at 1000 rpm. Samples were placed on a magnetic rack and supernatant was aspirated. Beads were washed with 125 μl 80% ethanol three times by resuspending. Beads were resuspended in 40 μl digest buffer (200 mM EPPS pH 8.5, 20 ng μl-1 LysC), then incubated overnight at 37 °C with constant agitation, before adding trypsin (400 ng) for an additional 6 h at 37 °C. The final concentration of peptide was ~1 μg/μl. Two replicates of each cell line were manually labeled with a set of TMTpro 16-plex reagents, and the mixture was incubated at room temperature for 1 h. The reaction was quenched with 0.5% hydroxylamine, before pooling beads and eluates into a single 2 ml tube and placing it on a magnetic rack to removed SP3 beads. The supernatant was collected after labeling, mixed, and peptide mixtures were desalted using C18 sep-pak cartridges (50 mg, Waters).

For phosphopeptide enrichment, PureCube magnetic beads were used with modified protocol^86^. In brief, peptides were dissolved in 80% ACN, 0.1% TFA and mixed with beads, shaking for 30 min. Phosphorylated peptides were eluted with 50% ACN, 2.5% NH_4_OH and then neutralized with 75% ACN, 10% formic acid. Elutes were desalted via Stage-tips prior to mass spectrometry analysis. Flow-throughs were fractionated using basic-pH reverse phase chromatography^87^. Briefly, peptides were resuspended in Buffer A (10 mM ammonium bicarbonate, 5% acetonitrile, pH 8) and separated on a linear gradient from 13% to 42% Buffer B (10 mM ammonium bicarbonate, 90% acetonitrile, pH 8) over an Agilent 300Extend C18 column using an Agilent 1260 HPLC equipped with wavelength detection at 214 nm and 254 nm). Fractionated peptides were desalted using Stage-tips prior to mass spectrometry analysis.

### Sample preparation for protein thermal stability assay

Thermal proteome profiling (TPP) by protein integral solubility assay (PISA) was performed as previously described^88^. Each PISA experiment was performed in triplicate.

For live-cell PISA treatment, A549 cells were grown to a density of 1 × 10^6^ cells/mL, isolated by centrifugation, and resuspended in fresh media to 6 × 10^6^ cells/mL to each well of a 24-well plate was added 500 uL of cell suspension and 1 mL of treatment (or vehicle) in media for a final cell concentration of 2 × 10^6^ cells/mL and a final treatment concentration of 10 µM. Cells were incubated with treatment for 1 h at room temperature. 1 mL from each incubated sample (2 × 10^6^ cells) was collected, washed once in PBS, and resuspended in PBS containing 10 µM treatment. For each treatment condition, 30 µL of sample was aliquoted into 10 wells of a PCR plate and subjected to a temperature gradient from 48 °C to 58 °C for 3 min, then allowed to cool to room temperature for 5 minutes. 30 µL of ice-cold lysis buffer (200 mM EPPS pH 7.2, 150 mM NaCl, Roche cOmplete protease inhibitor, 0.5% NP-40) was added to each PCR plate well. 50 µL from each temperature point of a single condition was collected and pooled and cells were allowed to lyse at 4 °C for 15 min. Lysate was spun at maximum speed for 2 h and the soluble fraction was collected for sample preparation for proteomic analysis.

For lysate PISA treatment, cells were resuspended in the lysis buffer as above and lysed by repeated aspiration through a 21-gauge needle. Lysate was centrifuged at maximum speed for 30 min at 4 °C. The soluble fraction was separated, and lysate protein content was determined by BCA. Lysate and treatment were combined in lysis buffer for a final protein concentration of 1 mg/mL and a treatment concentration of 10 µM and then incubated at room temperature for 15 min. For each treatment condition, 30 µL of sample was aliquoted into 10 wells of a PCR plate and subjected to a temperature gradient as above. 25 µL from each temperature point of a single condition was collected, pooled and centrifuged at maximum speed for 2 h. The soluble fraction was collected for sample preparation for proteomic analysis.

### Mass spectrometry data analysis for multiplexed quantitative proteomics

As previously described in ref. ^85^, Peptides were separated prior to MS/MS analysis using an Easy-nLC (Thermo) equipped with an in-house pulled fused silica capillary column with integrated emitter packed with Accucore C18 media (Thermo). Mass spectrometric analysis was carried out on an Orbitrap Eclipse (Thermo). For whole proteome profiling, separation was carried out with 90 min gradients from 98% Buffer A (5% ACN, 0.125% formic acid) to 28% Buffer B (90% ACN, 0.125% formic acid). Multiplexed analysis of samples was done using real-time search data acquisition^89^, based on canonical SPS-MS3 acquisition. Briefly, a survey MS1 scan was used to identify potential peptide precursors (R = 120000, Mass range: 400–2000 m/z, max Inject time: 50 ms, AGC: 200%, RF lens: 30%). The top 10 precursors were selected for fragmentation and analysis in the ion trap (Dynamic exclusion: 90 s at 10 ppm, CID collision energy: 35%, max inject time: 50 ms, AGC: 200%, scan rate: rapid, isolation width: 0.5 m/z). Real-time spectral matching was carried out using the Comet search algorithm^90^. If, and only if, a peptide was matched with high confidence, the instrument would then acquire an additional SPS-MS3 scan for quantification of relative abundances (R = 50000, HCD NCE: 45, max injection time: 150 ms).

For phosphopeptide profiling, separation was carried out with 90-minute gradients from 98% Buffer A (5% ACN, 0.125% formic acid) to 26% Buffer B (90% ACN, 0.125% formic acid). As previously described in ref. ^85^, multiplexed analysis of samples was done using canonical SPS-MS3 acquisition. Briefly, a survey MS1 scan was used to identify potential peptide precursors (R = 120,000; Mass range: 300–2000 m/z, max Inject time: 50 ms, AGC: 200%, RF lens: 30%). The top 10 precursors were selected for fragmentation and analysis in the ion trap (Dynamic exclusion: 90 s at 10 ppm, HCD NCE: 30, max inject time: 35 ms, AGC: 250%, scan rate: rapid, isolation width: 0.5 m/z). SPS-MS3 was carried out in the Orbitrap (R = 50,000; AGC: 250%, HCD NCE: 45, max injection time: 86 ms).

Raw spectral information was converted to mzXML format and monoisotopic masses were corrected using Monocle^91^, and spectra were matched using the Comet search algorithm compared against the Uniprot human database. TMTpro is a static modification at the N-terminus of peptides. The maximum missed cleavages allowed was set as 2. Peptides and proteins were filtered to a 1% false discovery rate using the rules of protein parsimony.

### Histone extraction and LC-MS/MS analysis for HDACi treatment

The histones were extracted and prepared for chemical derivatization and digestion as described previously in refs. ^92,93^. Briefly, lysine residues on histones were derivatized with the propionylation reagent (1:2 reagent:sample ratio) containing acetonitrile and propionic anhydride (3:1), with the solution pH adjusted to 8.0 using ammonium hydroxide. This propionylation reaction was performed twice and the samples were then dried using a speed vac. The derivatized histones were subsequently digested with trypsin at a 1:50 ratio (wt/wt) in 50 mM ammonium bicarbonate buffer at room temperature overnight. The N-termini of histone peptides were derivatized with the propionylation reagent twice and dried on speed vac^94^. The peptides were desalted with the self-packed C18 stage tip. After desalting, the purified peptides were dried and reconstituted in 0.1% formic acid. Peptide analysis was performed using a LC-MS/MS system consisting of a Vanquish Neo UHPLC coupled to an Orbitrap Exploris 240 (Thermo Scientific). The histone peptide samples were kept at 7 °C on sample trays during LC analysis. Separation of peptides was carried out on an Easy-Spray™ PepMap™ Neo nano-column (2 µm, C18, 75 µm X 150 mm) at room temperature with a mobile phase. The chromatography conditions consisted of a linear gradient from 2 to 32% solvent B (0.1% formic acid in 100% acetonitrile) in solvent A (0.1% formic acid in water) over 48 minutes, followed by 42 to 98% solvent B over 12 min, at a flow rate of 300 nL/min. The mass spectrometer was programmed for data-independent acquisition (DIA) where each acquisition cycle consisted of a full MS scan, 35 DIA MS/MS scans of 24 m/z isolation width starting from 295 m/z to reach 1100 m/z. Typically, full MS scans were acquired in the Orbitrap mass analyzer across 290–1100 m/z at a resolution of 60,000 in positive profile mode with an auto maximum injection time and an AGC target of 300%. MS/MS data from HCD fragmentation was collected in the Orbitrap. These scans typically used an NCE of 30, an AGC target of 1000%, and a maximum injection time of 60 ms. Histone MS data were analyzed with EpiProfile 2.0^95^.

### Transcriptomics

Data regarding the HDACi-induced transcriptomic response in A549 was obtained from the NCBI GEO database under sample GSM4150378^3^. Briefly, in this study, A549, MCF7, and K562 cells were treated with 4 increasing doses of a library of 188 small molecule compounds, including a subset of HDACi. The treated cells were subjected to high-throughput combinatorial indexing single-cell RNA-seq (sci-RNA-seq3). For this analysis, R package *monocle3* was used to analyze differentially expressed genes (DEGs) and for UMAP visualization^96–98^. For DEG analysis, the *celldataset* (cds) object was filtered to contain only A549 cells exposed to HDACi or vehicle control. For each HDACi, a generalized linear model was used to fit gene expression as a function of dose and replicate (quasi-Poisson regression) using *monocle3*’s *fit_models* function. This operation was performed on 7832 genes, determined as the union of genes expressed in 5% of cells per each inhibitor group. The p-values (Wald test) were FDR-corrected using the Benjamini-Hochberg method for multiple hypotheses, and the beta coefficients related to the contribution of dose were extracted and filtered as normalized beta coefficient > 0.05 and FDR < 5% for downstream comparison. Lastly, we performed batch integration^99^ and dimensional reduction^100^ of A549 cells treated with Abexinostat, Belinostat, Panobinostat, Trichostatin A, or vehicle to visualize cellular responses to HDACi.

### Western blot

A549 (CVCL_0023) cells were dispensed into tissue culture treated 6 well plates at a density of 40,000 cells per well in a volume of 2 ml. After 24 h, cells growing at less than 80% confluence were treated with 10 μM of belinostat, barasertib, and combination of belinostat and barasertib or 0.1% DMSO. After treatment, cells were incubated for 30 min at 37 °C before washing with 1 mL PBS per well. After all PBS was aspirated, cells were lysed with buffer containing 150 mM NaCl, 200 mM EPPS pH 8.5, 1% NP-40, 1% Triton X-100 and Roche protease inhibitor tablets. From each treatment, 20 μg of protein lysates was loaded onto a gel (Bolt™ Bis-Tris Plus Mini Protein Gels, 4–12%, 1.0 mm, WedgeWell). Proteins were then transferred to PVDF membrane using an iBlot 2 system. Phospho-Histone H3 (Ser10) Antibody (Cell signaling #9701), Aurora B/AIM1 (E3R8N) Rabbit mAb (Cell signaling #28711) and Phospho-Aurora A (Thr288)/Aurora B (Thr232)/Aurora C (Thr198) (D13A11) XP® Rabbit mAb (Cell signaling #2914) were used for western blot.

### Data analysis

Protein quantification tables containing normalized TMT abundance ratios were analyzed with python. Coefficient of variation (CV) between replicates was calculated and filtered (CV < 0.3) before being calculated relative to DMSO. Networks were imported from BioPlex 3.0^54,55^ and modified in Cytoscape^101^. Molecular docking of belinostat (PubChem: 6918638) to AURKB (PDB: 4AF3^102^) was performed using AutoDock 4.2 within AutoDockTools 1.5.7^103^. Docking was performed using 100 Genetic Algorithm runs with Lamarckian output, all other parameters were set to default parameters.

Software used as listed:

**Table:** 

Software	Version
Python	3.11.1
pandas	2.1.4
numpy	1.26.3
matplotlib	3.7.0
plotnine	0.12.4
scipy	1.11.3
statsmodels	0.14.0
PyComplexHeatmap	1.6.1
seaborn	0.13.0
gsepy	1.1.1
scikit-learn	1.3.1
Cytoscape	3.10.1
BioPlex	3.0
AutoDockTools	1.5.7
AutoDock	4.2

### Statistics and Reproducibility

Proteomic and phosphoproteomic screens were performed in duplicate biological replicates and are reported in the Supplementary Data. All other experiments were performed in biological triplicate. Statistical testing and sample sizes are specified for each experiment in their corresponding figure legend.

### Reporting summary

Further information on research design is available in the Nature Portfolio Reporting Summary linked to this article.

## Supplementary information

Supplementary Tables and Figures

Description of Additional Supplementary File

Supplementary Data 1

Supplementary Data 2

Supplementary Data 3

Supplementary Data 4

Supplementary Data 5

Supplementary Data 6

Reporting Summary

## Data Availability

For total proteomics and phosphoproteomics, 70 RAW files have been deposited to the ProteomeXchange Consortium via the PRIDE partner repository with the data set identifier PXD051206. Spectra were searched against the human Uniprot database (download v.05/2020). Numerical source data can be found in Supplementary Data 3 and 4.

## Code availability

The data viewer can be found at https://github.com/SchweppeLab/HDAC-Perturbation-DataViewer (DOI: 10.5281/zenodo.17575824). The data viewer was built in python using Streamlit and hosted through a docker container. Relevant information for starting the local server can be found in the GitHub repository README. The data viewer code is also deposited to Zenodo (10.5281/zenodo.17575824).

## References

[1] Chen, P.-H. et al. Metabolic Diversity in Human Non-Small Cell Lung Cancer Cells. *Mol. Cell* **76**, 838–851.e5 (2019). 31564558 10.1016/j.molcel.2019.08.028 PMC6898782

[2] Corsello, S. M. et al. Discovering the anticancer potential of non-oncology drugs by systematic viability profiling. *Nat. Cancer* **1**, 235–248 (2020). 32613204 10.1038/s43018-019-0018-6 PMC7328899

[3] Srivatsan, S. R. et al. Massively multiplex chemical transcriptomics at single-cell resolution. *Science* **367**, 45–51 (2020). 31806696 10.1126/science.aax6234 PMC7289078

[4] Maltz, E. & Wollman, R. Quantifying the phenotypic information in mRNA abundance. *Mol. Syst. Biol.* **18**, e11001 (2022). 35965452 10.15252/msb.202211001 PMC9376724

[5] Wang, D. et al. A deep proteome and transcriptome abundance atlas of 29 healthy human tissues. *Mol. Syst. Biol.* **15**, e8503 (2019). 30777892 10.15252/msb.20188503 PMC6379049

[6] Mitchell, D. C. et al. A proteome-wide atlas of drug mechanism of action. *Nat. Biotechnol*. 10.1038/s41587-022-01539-0 (2023). 10.1038/s41587-022-01539-0 PMC11069389 36593396

[7] Zecha, J. et al. Decrypting drug actions and protein modifications by dose- and time-resolved proteomics. *Science*10.1126/science.ade3925 (2023). 10.1126/science.ade3925 PMC7615311 36926954

[8] Begley, C. G. & Ellis, L. M. Raise standards for preclinical cancer research. *Nature* **483**, 531–533 (2012). 22460880 10.1038/483531a

[9] Raghavan, S. How inclusive are cell lines in preclinical engineered cancer models? *Dis. Model. Mech*. **15**, dmm049520 (2022). 10.1242/dmm.049520 PMC9187871 35642685

[10] Li, Y. & Seto, E. HDACs and HDAC Inhibitors in Cancer Development and Therapy. *Cold Spring Harb. Perspect. Med.* **6**, a026831 (2016). 27599530 10.1101/cshperspect.a026831 PMC5046688

[11] Zhang, Y. et al. Collateral lethality between HDAC1 and HDAC2 exploits cancer-specific NuRD complex vulnerabilities. *Nat. Struct. Mol. Biol.* **30**, 1160–1171 (2023). 37488358 10.1038/s41594-023-01041-4 PMC10529074

[12] Yoshida, M., Kijima, M., Akita, M. & Beppu, T. Potent and specific inhibition of mammalian histone deacetylase both in vivo and in vitro by trichostatin A. *J. Biol. Chem.* **265**, 17174–17179 (1990). 2211619

[13] Mann, B. S., Johnson, J. R., Cohen, M. H., Justice, R. & Pazdur, R. FDA Approval Summary: Vorinostat for Treatment of Advanced Primary Cutaneous T-Cell Lymphoma. *Oncologist* **12**, 1247–1252 (2007). 17962618 10.1634/theoncologist.12-10-1247

[14] McDermott, J. & Jimeno, A. Belinostat for the treatment of peripheral T-cell lymphomas. *Drugs Today Barc. Spain* **50**, 337–345 (2014). 10.1358/dot.2014.50.5.2138703 24918834

[15] Richardson, P. G. et al. Panobinostat: a novel pan-deacetylase inhibitor for the treatment of relapsed or relapsed and refractory multiple myeloma. *Expert Rev. Anticancer Ther.* **15**, 737–748 (2015). 26051506 10.1586/14737140.2015.1047770

[16] Mamdani, H. & Jalal, S. I. Histone Deacetylase Inhibition in Non-small Cell Lung Cancer: Hype or Hope? *Front. Cell Dev. Biol*. **8**, 582370 (2020). 10.3389/fcell.2020.582370 PMC7581936 33163495

[17] Traynor, A. M. et al. Vorinostat (NSC# 701852) in Patients with Relapsed Non-small Cell Lung Cancer: A Wisconsin Oncology Network Phase II Study. *J. Thorac. Oncol.* **4**, 522–526 (2009). 19347984 10.1097/jto.0b013e3181952478 PMC3050710

[18] Reid, T. et al. Phase II trial of the histone deacetylase inhibitor pivaloyloxymethyl butyrate (Pivanex, AN-9) in advanced non-small cell lung cancer. *Lung Cancer* **45**, 381–386 (2004). 15301879 10.1016/j.lungcan.2004.03.002

[19] Schrump, D. S. et al. Clinical and Molecular Responses in Lung Cancer Patients Receiving Romidepsin. *Clin. Cancer Res.* **14**, 188–198 (2008). 18172270 10.1158/1078-0432.CCR-07-0135

[20] Miyanaga, A. et al. Antitumor activity of histone deacetylase inhibitors in non-small cell lung cancer cells: development of a molecular predictive model. *Mol. Cancer Ther.* **7**, 1923–1930 (2008). 18606719 10.1158/1535-7163.MCT-07-2140

[21] Richon, V. M. et al. Second generation hybrid polar compounds are potent inducers of transformed cell differentiation. *Proc. Natl. Acad. Sci. USA.* **93**, 5705–5708 (1996). 8650156 10.1073/pnas.93.12.5705 PMC39124

[22] Xiao, H. & Hasegawa, T. & Isobe, K. Both Sp1 and Sp3 are responsible for p21waf1 promoter activity induced by histone deacetylase inhibitor in NIH3T3 cells. *J. Cell. Biochem.* **73**, 291–302 (1999). 10321829

[23] Vrana, J. A. et al. Induction of apoptosis in U937 human leukemia cells by suberoylanilide hydroxamic acid (SAHA) proceeds through pathways that are regulated by Bcl-2/Bcl-XL, c-Jun, and p21CIP1, but independent of p53. *Oncogene* **18**, 7016–7025 (1999). 10597302 10.1038/sj.onc.1203176

[24] Cao, X. X., Mohuiddin, I., Ece, F., McConkey, D. J. & Smythe, W. R. Histone Deacetylase Inhibitor Downregulation of bcl-xl Gene Expression Leads to Apoptotic Cell Death in Mesothelioma. *Am. J. Respir. Cell Mol. Biol.* **25**, 562–568 (2001). 11713097 10.1165/ajrcmb.25.5.4539

[25] Fandy, T. E. & Srivastava, R. K. Trichostatin A sensitizes TRAIL-resistant myeloma cells by downregulation of the antiapoptotic Bcl-2 proteins. *Cancer Chemother. Pharmacol.* **58**, 471–477 (2006). 16435155 10.1007/s00280-005-0184-3

[26] Liu, Y.-L. et al. Autophagy potentiates the anti-cancer effects of the histone deacetylase inhibitors in hepatocellular carcinoma. *Autophagy* **6**, 1057–1065 (2010). 20962572 10.4161/auto.6.8.13365

[27] Shulak, L. et al. Histone Deacetylase Inhibitors Potentiate Vesicular Stomatitis Virus Oncolysis in Prostate Cancer Cells by Modulating NF-κB-Dependent Autophagy. *J. Virol.* **88**, 2927–2940 (2014). 24371063 10.1128/JVI.03406-13 PMC3958113

[28] Lechner, S. et al. Target deconvolution of HDAC pharmacopoeia reveals MBLAC2 as common off-target. *Nat. Chem. Biol.* **18**, 812–820 (2022). 35484434 10.1038/s41589-022-01015-5 PMC9339481

[29] Tsherniak, A. et al. Defining a Cancer Dependency Map. *Cell* **170**, 564–576.e16 (2017). 28753430 10.1016/j.cell.2017.06.010 PMC5667678

[30] Nusinow, D. P. et al. Quantitative Proteomics of the Cancer. *Cell Line Encycl. Cell* **180**, 387–402.e16 (2020). 10.1016/j.cell.2019.12.023 PMC7339254 31978347

[31] Eckert, S. et al. Decrypting the molecular basis of cellular drug phenotypes by dose-resolved expression proteomics. *Nat. Biotechnol*. 1–10 10.1038/s41587-024-02218-y (2024). 10.1038/s41587-024-02218-y PMC11919725 38714896

[32] Shanmugam, G., Rakshit, S. & Sarkar, K. HDAC inhibitors: Targets for tumor therapy, immune modulation and lung diseases. *Transl. Oncol.* **16**, 101312 (2022). 34922087 10.1016/j.tranon.2021.101312 PMC8688863

[33] Buggy, J. J. et al. CRA-024781: a novel synthetic inhibitor of histone deacetylase enzymes with antitumor activity in vitro and in vivo. *Mol. Cancer Ther.* **5**, 1309–1317 (2006). 16731764 10.1158/1535-7163.MCT-05-0442

[34] Kelly, W. K. et al. Phase I Study of an Oral Histone Deacetylase Inhibitor, Suberoylanilide Hydroxamic Acid, in Patients With Advanced Cancer. *J. Clin. Oncol.* **23**, 3923–3931 (2005). 15897550 10.1200/JCO.2005.14.167 PMC1855284

[35] Nukaga, S. et al. Amplification of EGFR Wild-Type Alleles in Non-Small Cell Lung Cancer Cells Confers Acquired Resistance to Mutation-Selective EGFR Tyrosine Kinase Inhibitors. *Cancer Res* **77**, 2078–2089 (2017). 28202511 10.1158/0008-5472.CAN-16-2359

[36] Mader, M. et al. Imidazolyl benzimidazoles and imidazo[4,5-b]pyridines as potent p38alpha MAP kinase inhibitors with excellent in vivo antiinflammatory properties. *Bioorg. Med. Chem. Lett.* **18**, 179–183 (2008). 18039577 10.1016/j.bmcl.2007.10.106

[37] Uhlén, M. et al. Tissue-based map of the human proteome. *Science* **347**, 1260419 (2015). 25613900 10.1126/science.1260419

[38] Caslini, C., Hong, S., Ban, Y. J., Chen, X. S. & Ince, T. A. HDAC7 regulates histone 3 lysine 27 acetylation and transcriptional activity at super-enhancer-associated genes in breast cancer stem cells. *Oncogene* **38**, 6599–6614 (2019). 31375747 10.1038/s41388-019-0897-0

[39] Ma, C. & D’Mello, S. R. Neuroprotection by Histone Deacetylase-7 (HDAC7) Occurs by Inhibition of c-jun Expression through a Deacetylase-independent Mechanism. *J. Biol. Chem.* **286**, 4819–4828 (2011). 21118817 10.1074/jbc.M110.146860 PMC3039335

[40] Chen, S. et al. The PZP Domain of AF10 Senses Unmodified H3K27 to Regulate DOT1L-Mediated Methylation of H3K79. *Mol. Cell* **60**, 319–327 (2015). 26439302 10.1016/j.molcel.2015.08.019 PMC4609290

[41] Xie, Y. et al. SETD2 loss perturbs the kidney cancer epigenetic landscape to promote metastasis and engenders actionable dependencies on histone chaperone complexes. *Nat. Cancer* **3**, 188–202 (2022). 35115713 10.1038/s43018-021-00316-3 PMC8885846

[42] Nichol, J. N., Dupéré-Richer, D., Ezponda, T., Licht, J. D. & Miller, W. H. Chapter Three - H3K27 Methylation: A Focal Point of Epigenetic Deregulation in Cancer. in *Advances in Cancer Research* (eds Tew, K. D. & Fisher, P. B.) vol. 131, 59–95 (Academic Press, 2016). 10.1016/bs.acr.2016.05.001 PMC5325795 27451124

[43] Adhikary, S. et al. Selective Recognition of H3.1K36 Dimethylation/H4K16 Acetylation Facilitates the Regulation of All-trans-retinoic Acid (ATRA)-responsive Genes by Putative Chromatin Reader ZMYND8. *J. Biol. Chem.* **291**, 2664–2681 (2016). 26655721 10.1074/jbc.M115.679985 PMC4742736

[44] Alvarez, F. et al. Sequential Establishment of Marks on Soluble Histones H3 and H4. *J. Biol. Chem.* **286**, 17714–17721 (2011). 21454524 10.1074/jbc.M111.223453 PMC3093847

[45] Cong, P. et al. The transcriptional regulation role of BRD7 by binding to acetylated histone through bromodomain. *J. Cell. Biochem.* **97**, 882–892 (2006). 16265664 10.1002/jcb.20645

[46] Li, N. et al. ZMYND8 reads the dual histone mark H3K4me1-H3K14ac to antagonize the expression of metastasis-linked genes. *Mol. Cell* **63**, 470–484 (2016). 27477906 10.1016/j.molcel.2016.06.035 PMC4975651

[47] Mukherjee, S. et al. A novel role of tumor suppressor ZMYND8 in inducing differentiation of breast cancer cells through its dual-histone binding function. *J. Biosci.* **45**, 2 (2020). 31965980

[48] Chugunov, A. O. et al. Conserved Structure and Evolution of DPF Domain of PHF10—The Specific Subunit of PBAF Chromatin Remodeling Complex. *Int. J. Mol. Sci.* **22**, 11134 (2021). 34681795 10.3390/ijms222011134 PMC8538644

[49] Liu, X. et al. ASF1B promotes cervical cancer progression through stabilization of CDK9. *Cell Death Dis.* **11**, 1–15 (2020). 32848135 10.1038/s41419-020-02872-5 PMC7449975

[50] Jiang, X. et al. DACT3 is an epigenetic regulator of Wnt/beta-catenin signaling in colorectal cancer and is a therapeutic target of histone modifications. *Cancer Cell* **13**, 529–541 (2008). 18538736 10.1016/j.ccr.2008.04.019 PMC2577847

[51] Richon, V. M., Sandhoff, T. W., Rifkind, R. A. & Marks, P. A. Histone deacetylase inhibitor selectively induces p21WAF1 expression and gene-associated histone acetylation. *Proc. Natl. Acad. Sci.* **97**, 10014–10019 (2000). 10954755 10.1073/pnas.180316197 PMC27656

[52] Siavoshian, S. et al. Butyrate and trichostatin A effects on the proliferation/differentiation of human intestinal epithelial cells: induction of cyclin D3 and p21 expression. *Gut* **46**, 507–514 (2000). 10716680 10.1136/gut.46.4.507 PMC1727889

[53] Majumdar, G., Adris, P., Bhargava, N., Chen, H. & Raghow, R. Pan-histone deacetylase inhibitors regulate signaling pathways involved in proliferative and pro-inflammatory mechanisms in H9c2 cells. *BMC Genomics* **13**, 709 (2012). 23249388 10.1186/1471-2164-13-709 PMC3561284

[54] Huttlin, E. L. et al. Dual proteome-scale networks reveal cell-specific remodeling of the human interactome. *Cell* **184**, 3022–3040.e28 (2021). 33961781 10.1016/j.cell.2021.04.011 PMC8165030

[55] Schweppe, D. K., Huttlin, E. L., Harper, J. W. & Gygi, S. P. BioPlex Display: An Interactive Suite for Large-Scale AP–MS Protein–Protein Interaction Data. *J. Proteome Res.* **17**, 722–726 (2018). 29054129 10.1021/acs.jproteome.7b00572 PMC7029486

[56] Cho, Y., Sloutsky, R., Naegle, K. M. & Cavalli, V. Injury-Induced HDAC5 Nuclear Export Is Essential for Axon Regeneration. *Cell* **155**, 894–908 (2013). 24209626 10.1016/j.cell.2013.10.004 PMC3987749

[57] Liberzon, A. et al. The Molecular Signatures Database (MSigDB) hallmark gene set collection. *Cell Syst.* **1**, 417–425 (2015). 26771021 10.1016/j.cels.2015.12.004 PMC4707969

[58] van der Meer, D. et al. Cell Model Passports—a hub for clinical, genetic and functional datasets of preclinical cancer models. *Nucleic Acids Res* **47**, D923–D929 (2019). 30260411 10.1093/nar/gky872 PMC6324059

[59] Gaetani, M. et al. Proteome Integral Solubility Alteration: A High-Throughput Proteomics Assay for Target Deconvolution. *J. Proteome Res.* **18**, 4027–4037 (2019). 31545609 10.1021/acs.jproteome.9b00500

[60] Van Vranken, J. G. et al. Large-scale characterization of drug mechanism of action using proteome-wide thermal shift assays. *eLife* **13**, RP95595 (2024). 39526730 10.7554/eLife.95595 PMC11554310

[61] Blackledge, N. P. & Klose, R. J. The molecular principles of gene regulation by Polycomb repressive complexes. *Nat. Rev. Mol. Cell Biol.* **22**, 815–833 (2021). 34400841 10.1038/s41580-021-00398-y PMC7612013

[62] Hakimi, M.-A., Dong, Y., Lane, W. S., Speicher, D. W. & Shiekhattar, R. A Candidate X-linked Mental Retardation Gene Is a Component of a New Family of Histone Deacetylase-containing Complexes *. *J. Biol. Chem.* **278**, 7234–7239 (2003). 12493763 10.1074/jbc.M208992200

[63] Leung, J. W. C. et al. ZMYM3 regulates BRCA1 localization at damaged chromatin to promote DNA repair. *Genes Dev.* **31**, 260–274 (2017). 28242625 10.1101/gad.292516.116 PMC5358723

[64] Zhang, J. et al. Src-Family Kinases Are Activated in Non-Small Cell Lung Cancer and Promote the Survival of Epidermal Growth Factor Receptor-Dependent Cell Lines. *Am. J. Pathol.* **170**, 366–376 (2007). 17200208 10.2353/ajpath.2007.060706 PMC1762707

[65] Hirsch, C. L., Smith-Windsor, E. L. & Bonham, K. Src family kinase members have a common response to histone deacetylase inhibitors in human colon cancer cells. *Int. J. Cancer* **118**, 547–554 (2006). 16094635 10.1002/ijc.21383

[66] Golkowski, M. et al. Multiplexed kinase interactome profiling quantifies cellular network activity and plasticity. *Mol. Cell* **83**, 803–818.e8 (2023). 36736316 10.1016/j.molcel.2023.01.015 PMC10072906

[67] Göransson, O. et al. Regulation of the polarity kinases PAR-1/MARK by 14-3-3 interaction and phosphorylation. *J. Cell Sci.* **119**, 4059–4070 (2006). 16968750 10.1242/jcs.03097

[68] Segal, D. et al. A central chaperone-like role for 14-3-3 proteins in human cells. *Mol. Cell* **83**, 974–993.e15 (2023). 36931259 10.1016/j.molcel.2023.02.018

[69] Shen, C. et al. The 14-3-3ζ-c-Src-integrin-β3 complex is vital for platelet activation. *Blood* **136**, 974–988 (2020). 32584951 10.1182/blood.2019002314

[70] Li, X., Song, S., Liu, Y., Ko, S.-H. & Kao, H.-Y. Phosphorylation of the Histone Deacetylase 7 Modulates Its Stability and Association with 14-3-3 Proteins *. *J. Biol. Chem.* **279**, 34201–34208 (2004). 15166223 10.1074/jbc.M405179200

[71] Nishino, T. G. et al. 14-3-3 regulates the nuclear import of class IIa histone deacetylases. *Biochem. Biophys. Res. Commun.* **377**, 852–856 (2008). 18952052 10.1016/j.bbrc.2008.10.079

[72] Wiredja, D. D., Koyutürk, M. & Chance, M. R. The KSEA App: a web-based tool for kinase activity inference from quantitative phosphoproteomics. *Bioinformatics* **33**, 3489–3491 (2017). 28655153 10.1093/bioinformatics/btx415 PMC5860163

[73] Mann, J. et al. Non-canonical BAD activity regulates breast cancer cell and tumor growth via 14-3-3 binding and mitochondrial metabolism. *Oncogene* **38**, 3325–3339 (2019). 30635657 10.1038/s41388-018-0673-6 PMC6756016

[74] Polzien, L., Baljuls, A., Albrecht, M., Hekman, M. & Rapp, U. R. BAD Contributes to RAF-mediated Proliferation and Cooperates with B-RAF-V600E in Cancer Signaling*. *J. Biol. Chem.* **286**, 17934–17944 (2011). 21317286 10.1074/jbc.M110.177345 PMC3093868

[75] Datta, S. R. et al. 14-3-3 Proteins and Survival Kinases Cooperate to Inactivate BAD by BH3 Domain Phosphorylation. *Mol. Cell* **6**, 41–51 (2000). 10949026

[76] Tan, Y., Demeter, M. R., Ruan, H. & Comb, M. J. BAD Ser-155 Phosphorylation Regulates BAD/Bcl-XL Interaction and Cell Survival*. *J. Biol. Chem.* **275**, 25865–25869 (2000). 10837486 10.1074/jbc.M004199200

[77] Poll, B. G. et al. A resource database for protein kinase substrate sequence-preference motifs based on large-scale mass spectrometry data. *Cell Commun. Signal. CCS* **22**, 137 (2024). 38374071 10.1186/s12964-023-01436-2 PMC10875805

[78] LaBaer, J. et al. New functional activities for the p21 family of CDK inhibitors. *Genes Dev.* **11**, 847–862 (1997). 9106657 10.1101/gad.11.7.847

[79] Bockstaele, L. et al. Regulated Activating Thr172 Phosphorylation of Cyclin-Dependent Kinase 4(CDK4): Its Relationship with Cyclins and CDK “Inhibitors”. *Mol. Cell. Biol.* **26**, 5070–5085 (2006). 16782892 10.1128/MCB.02006-05 PMC1489149

[80] Sawai, C. M. et al. Therapeutic Targeting of the Cyclin D3:CDK4/6 Complex in T Cell Leukemia. *Cancer Cell* **22**, 452–465 (2012). 23079656 10.1016/j.ccr.2012.09.016 PMC3493168

[81] Leon, C. et al. Lack of racial and ethnic diversity in lung cancer cell lines contributes to lung cancer health disparities. *Front. Oncol*. **13**, 1187585 (2023). 10.3389/fonc.2023.1187585 PMC10651223 38023251

[82] Ghandi, M. et al. Next-generation characterization of the Cancer Cell Line Encyclopedia. *Nature* **569**, 503–508 (2019). 31068700 10.1038/s41586-019-1186-3 PMC6697103

[83] Papavassiliou, A. G. & Musti, A. M. The Multifaceted Output of c-Jun Biological Activity: Focus at the Junction of CD8 T Cell Activation and Exhaustion. *Cells* **9**, 2470 (2020). 33202877 10.3390/cells9112470 PMC7697663

[84] Jasencakova, Z. et al. Replication Stress Interferes with Histone Recycling and Predeposition Marking of New Histones. *Mol. Cell* **37**, 736–743 (2010). 20227376 10.1016/j.molcel.2010.01.033

[85] Edman, N. I. et al. Modulation of FGF pathway signaling and vascular differentiation using designed oligomeric assemblies. *Cell*. **11**,187 (2024). 10.1016/j.cell.2024.05.025 PMC11246234 38861993

[86] Leutert, M., Rodríguez-Mias, R. A., Fukuda, N. K. & Villén, J. R2-P2 rapid-robotic phosphoproteomics enables multidimensional cell signaling studies. *Mol. Syst. Biol.* **15**, e9021 (2019). 31885202 10.15252/msb.20199021 PMC6920700

[87] Navarrete-Perea, J., Yu, Q., Gygi, S. P. & Paulo, J. A. Streamlined Tandem Mass Tag (SL-TMT) Protocol: An Efficient Strategy for Quantitative (Phospho)proteome Profiling Using Tandem Mass Tag-Synchronous Precursor Selection-MS3. *J. Proteome Res.* **17**, 2226–2236 (2018). 29734811 10.1021/acs.jproteome.8b00217 PMC5994137

[88] Van Vranken, J. G. et al. Large-scale characterization of drug mechanism of action using proteome-wide thermal shift assays. *bioRxiv* 2024.01.26.577428 10.1101/2024.01.26.577428 (2024). 10.7554/eLife.95595 PMC11554310 39526730

[89] Schweppe, D. K. et al. Full-Featured, Real-Time Database Searching Platform Enables Fast and Accurate Multiplexed Quantitative Proteomics. *J. Proteome Res.* **19**, 2026–2034 (2020). 32126768 10.1021/acs.jproteome.9b00860 PMC7295121

[90] Eng, J. K., Jahan, T. A. & Hoopmann, M. R. Comet: An open-source MS/MS sequence database search tool. *PROTEOMICS* **13**, 22–24 (2013). 23148064 10.1002/pmic.201200439

[91] Rad, R. et al. Improved Monoisotopic Mass Estimation for Deeper Proteome Coverage. *J. Proteome Res.* **20**, 591–598 (2021). 33190505 10.1021/acs.jproteome.0c00563 PMC12204116

[92] Bhanu, N. V., Sidoli, S. & Garcia, B. A. A Workflow for Ultra-rapid Analysis of Histone Post-translational Modifications with Direct-injection Mass Spectrometry. *Bio-Protoc.* **10**, e3756 (2020). 33659415 10.21769/BioProtoc.3756 PMC7842335

[93] Sidoli, S., Bhanu, N. V., Karch, K. R., Wang, X. & Garcia, B. A. Complete Workflow for Analysis of Histone Post-translational Modifications Using Bottom-up Mass Spectrometry: From Histone Extraction to Data Analysis. *J. Vis. Exp. JoVE* 54112. 10.3791/54112 (2016). 10.3791/54112 PMC4927705 27286567

[94] Searfoss, R. M., Karki, R., Lin, Z., Robison, F. & Garcia, B. A. An Optimized and High-Throughput Method for Histone Propionylation and Data-Independent Acquisition Analysis for the Identification and Quantification of Histone Post-translational Modifications. *J. Am. Soc. Mass Spectrom.* **34**, 2508–2517 (2023). 37853520 10.1021/jasms.3c00223 PMC11017827

[95] Yuan, Z.-F. et al. EpiProfile Quantifies Histone Peptides With Modifications by Extracting Retention Time and Intensity in High-resolution Mass Spectra*. *Mol. Cell. Proteom.* **14**, 1696–1707 (2015). 10.1074/mcp.M114.046011 PMC4458730 25805797

[96] Trapnell, C. et al. The dynamics and regulators of cell fate decisions are revealed by pseudotemporal ordering of single cells. *Nat. Biotechnol.* **32**, 381–386 (2014). 24658644 10.1038/nbt.2859 PMC4122333

[97] Qiu, X. et al. Reversed graph embedding resolves complex single-cell trajectories. *Nat. Methods* **14**, 979–982 (2017). 28825705 10.1038/nmeth.4402 PMC5764547

[98] Cao, J. et al. The single-cell transcriptional landscape of mammalian organogenesis. *Nature* **566**, 496–502 (2019). 30787437 10.1038/s41586-019-0969-x PMC6434952

[99] Haghverdi, L., Lun, A. T. L., Morgan, M. D. & Marioni, J. C. Batch effects in single-cell RNA sequencing data are corrected by matching mutual nearest neighbours. *Nat. Biotechnol.* **36**, 421–427 (2018). 29608177 10.1038/nbt.4091 PMC6152897

[100] McInnes, L., Healy, J., Saul, N. & Großberger, L. UMAP: Uniform Manifold Approximation and Projection. *J. Open Source Softw.* **3**, 861 (2018).

[101] Shannon, P. et al. Cytoscape: a software environment for integrated models of biomolecular interaction networks. *Genome Res* **13**, 2498–2504 (2003). 14597658 10.1101/gr.1239303 PMC403769

[102] Elkins, J. M., Santaguida, S., Musacchio, A. & Knapp, S. Crystal structure of human aurora B in complex with INCENP and VX-680. *J. Med. Chem.* **55**, 7841–7848 (2012). 22920039 10.1021/jm3008954 PMC3621106

[103] Morris, G. M. et al. AutoDock4 and AutoDockTools4: Automated docking with selective receptor flexibility. *J. Comput. Chem.* **30**, 2785–2791 (2009). 19399780 10.1002/jcc.21256 PMC2760638

