# Supplementary material for: Defining the heterogeneous molecular landscape of lung cancer cell responses to epigenetic inhibition
Source: Commun Biol. 2026 Jan 28;9:176. doi: 10.1038/s42003-025-09455-0 (PMC12876866; doi:10.1038/s42003-025-09455-0)
Supplement: Supplementary file 2 — Description of Additional Supplementary File [file 42003_2025_9455_MOESM2_ESM.pdf]

Description of Additional Supplementary Files

File name: Supplementary Data 1

Description: Normalized total protein abundance changes data

File name: Supplementary Data 2

Description: Normalized phosphosite abundance changes data

File name: Supplementary Data 3

Description: Source data for main figures

File name: Supplementary Data 4

Description: Source data for supplementary figures

File name: Supplementary Data 5

Description: Hallmark, Go and KEGG pathway enrichment results of proteins with abundance changes

File name: Supplementary Data 6

Description: Pearson correlation table of HDACs with proteins that were quantified in all cell line-by-drug samples.
