# Supplementary material for: Defining the heterogeneous molecular landscape of lung cancer cell responses to epigenetic inhibition
Source: Commun Biol. 2026 Jan 28;9:176. doi: 10.1038/s42003-025-09455-0 (PMC12876866; doi:10.1038/s42003-025-09455-0)
Supplement: Supplementary file 9 — Reporting Summary [file 42003_2025_9455_MOESM9_ESM.pdf]

nature portfolio

Corresponding author(s): Devin K. Shweppe

Last updated by author(s): Dec 2, 2025

Reporting Summary

Nature Portfolio wishes to improve the reproducibility of the work that we publish. This form provides structure for consistency and transparency in reporting. For further information on Nature Portfolio policies, see our Editorial Policies and the Editorial Policy Checklist.

Statistics

For all statistical analyses, confirm that the following items are present in the figure legend, table legend, main text, or Methods section.

n/a	Confirmed	
☐	☒	The exact sample size (*n*) for each experimental group/condition, given as a discrete number and unit of measurement
☐	☒	A statement on whether measurements were taken from distinct samples or whether the same sample was measured repeatedly
☒	☐	The statistical test(s) used AND whether they are one- or two-sided<br>*Only common tests should be described solely by name; describe more complex techniques in the Methods section.*
☐	☒	A description of all covariates tested
☒	☐	A description of any assumptions or corrections, such as tests of normality and adjustment for multiple comparisons
☐	☒	A full description of the statistical parameters including central tendency (e.g. means) or other basic estimates (e.g. regression coefficient) AND variation (e.g. standard deviation) or associated estimates of uncertainty (e.g. confidence intervals)
☒	☐	For null hypothesis testing, the test statistic (e.g. *F*, *t*, *r*) with confidence intervals, effect sizes, degrees of freedom and *P* value noted<br>*Give P values as exact values whenever suitable.*
☒	☐	For Bayesian analysis, information on the choice of priors and Markov chain Monte Carlo settings
☒	☐	For hierarchical and complex designs, identification of the appropriate level for tests and full reporting of outcomes
☐	☒	Estimates of effect sizes (e.g. Cohen's *d*, Pearson's *r*), indicating how they were calculated

Our web collection on statistics for biologists contains articles on many of the points above.

Software and code

Policy information about availability of computer code

Data collection	Raw spectral information was converted to mzXML format using Monocle (Rad et al., 2021), and spectra were matched using the Comet search algorithm compared against the Uniprot human database.
Data analysis	Python<br>3.11.1<br>pandas<br>2.1.4<br>numpy<br>1.26.3<br>matplotlib<br>3.7.0<br>plotnine<br>0.12.4<br>scipy<br>1.11.3<br>statsmodels<br>0.14.0<br>PyComplexHeatmap<br>1.6.1<br>seaborn<br>0.13.0

gsepy
1.1.1
scikit-learn
1.3.1
Cytoscape
3.10.1
BioPlex
3.0

For manuscripts utilizing custom algorithms or software that are central to the research but not yet described in published literature, software must be made available to editors and reviewers. We strongly encourage code deposition in a community repository (e.g. GitHub). See the Nature Portfolio guidelines for submitting code & software for further information.

Data

Policy information about availability of data

All manuscripts must include a data availability statement. This statement should provide the following information, where applicable:

- Accession codes, unique identifiers, or web links for publicly available datasets
- A description of any restrictions on data availability
- For clinical datasets or third party data, please ensure that the statement adheres to our policy

For total proteomics and phosphoproteomics, 70 .RAW files have been deposited to the ProteomeXchange Consortium via the PRIDE partner repository with the data set identifier PXD051206. Spectra were searched against the human Uniprot database (download v.05/2020).

Research involving human participants, their data, or biological material

Policy information about studies with human participants or human data. See also policy information about sex, gender (identity/presentation), and sexual orientation and race, ethnicity and racism.

Reporting on sex and gender	NA
Reporting on race, ethnicity, or other socially relevant groupings	NA
Population characteristics	NA
Recruitment	NA
Ethics oversight	NA

Note that full information on the approval of the study protocol must also be provided in the manuscript.

Field-specific reporting

Please select the one below that is the best fit for your research. If you are not sure, read the appropriate sections before making your selection.

☒ Life sciences ☐ Behavioural & social sciences ☐ Ecological, evolutionary & environmental sciences

For a reference copy of the document with all sections, see nature.com/documents/nr-reporting-summary-flat.pdf

Life sciences study design

All studies must disclose on these points even when the disclosure is negative.

Sample size	n=2 biological replicates per treatment group, 35 cell line by drug treatment groups based on Mitchell et al. 2023 (Nat Biotech)
Data exclusions	Proteins with coefficient of variation between replicates larger than 30% were excluded
Replication	n=2 biological replicates per treatment group, 35 cell line by drug treatment groups based on Mitchell et al. 2023 (Nat Biotech)
Randomization	NA
Blinding	NA

Reporting for specific materials, systems and methods

We require information from authors about some types of materials, experimental systems and methods used in many studies. Here, indicate whether each material, system or method listed is relevant to your study. If you are not sure if a list item applies to your research, read the appropriate section before selecting a response.

Materials & experimental systems

n/a	Involved in the study
☐	☒ Antibodies
☐	☒ Eukaryotic cell lines
☒	☐ Palaeontology and archaeology
☒	☐ Animals and other organisms
☒	☐ Clinical data
☒	☐ Dual use research of concern
☒	☐ Plants

Methods

n/a	Involved in the study
☒	☐ ChIP-seq
☒	☐ Flow cytometry
☒	☐ MRI-based neuroimaging

Antibodies

Antibodies used	Cell signaling Phospho-Histone H3 (Ser10) Antibody #9701;<br>Cell signaling Aurora B/AIM1 (E3R8N) Rabbit mAb #28711T;<br>Cell signaling Phospho-Aurora A (Thr288)/Aurora B (Thr232)/Aurora C (Thr198) (D13A11) XP® Rabbit mAb #2914T
Validation	Validation are done by the Cell signaling, statements are listed on their website as below:<br>Polyclonal antibodies are produced by immunizing animals with a synthetic phosphopeptide corresponding to residues surrounding Ser10 of human histone H3. Antibodies are purified by protein A and peptide affinity chromatography.<br>Monoclonal antibody is produced by immunizing animals with a synthetic peptide corresponding to residues near the amino terminus of human Auroro B/AIM1 protein.<br>Monoclonal antibody is produced by immunizing animals with a synthetic peptide corresponding to residues surrounding Thr232 of human Aurora B.

Eukaryotic cell lines

Policy information about cell lines and Sex and Gender in Research

Cell line source(s)	H292: ATCC CRL-1848<br>A549: ATCC CCL-185<br>PC9: RCB RCB4455<br>HCT116: ATCC CCL-247<br>PSC1: A gift from Dr. Steven Gygi's Lab
Authentication	None of the cell line used are authenticated
Mycoplasma contamination	Cell lines were tested negative for mycoplasma contamination
Commonly misidentified lines<br>(See ICLAC register)	NA

Plants

Seed stocks	NA
Novel plant genotypes	NA
Authentication	NA
